# Intelligent rendering optimization for animated scenes using reinforcement learning and computer vision

**DOI:** 10.1371/journal.pone.0361393

**Published:** 2026-11-10

**Authors:** Xiaoyan Li, Qiang Dai, Li Jia

**Affiliations:** 1 School of Animation and Digital Arts, Communication University of Shanxi, Jinzhong, Shanxi, China; 2 Department of Art and Design, Shanxi Polytechnic College, Taiyuan, Shanxi, China; University of Electronic Science and Technology of China, CHINA

## Abstract

Real-time rendering of complex animated scenes is computationally intensive and often struggles to balance visual fidelity and performance in dynamic or densely populated environments, as conventional pipelines lack adaptive resource optimization, leading to reduced frame rates and degraded visual quality. This research proposes an intelligent rendering framework integrating Computer Vision (CV) and Reinforcement Learning (RL) through the Adaptive Seeker–Twin Delayed Deep Deterministic Policy Gradient (AdapS-Twin D3PG) algorithm. The ATD-12 dataset consists of 10,000 collected animation image triplets designed for video frame interpolation and motion analysis tasks, and is utilized for training and evaluation. To enhance data quality, Savitzky–Golay filtering is applied to smooth motion trajectories while preserving essential temporal patterns, and Min–Max normalization standardizes frame color and intensity values for consistent input representation. Feature extraction using Histogram of Oriented Gradients (HOG) captures structural and shape-based information, enabling the RL agent to identify high-priority regions for selective rendering refinement. The proposed AdapS-Twin D3PG dynamically adjusts rendering parameters, refining critical regions while coarsely processing less significant areas to optimize computational efficiency. Experimental results from the Python-implemented framework demonstrate that the proposed AdapS-Twin D3PG model achieves superior quantitative performance with Accuracy (95.48), Recall (93.72), F1 Score (93.94), and AUC (96.82). The system also delivers enhanced visual reconstruction quality, evidenced by a PSNR of 39.4 dB and a low FID of 13.8, while maintaining an efficient rendering time of 4.2 seconds, thereby confirming its effectiveness in optimizing both visual fidelity and computational efficiency for real-time animated scene rendering.

## Introduction

The emergence of artificial intelligence methods specifically RL and CV have revolutionized the animated scene generation in many ways. Conventional animation projectors largely rely on hand-drawn guidelines, pro-programmed trajectories and manual control. Although it is possible to exercise very fine control over the behavior and motion of characters using these methods, it usually reduces flexibility and realism in dynamic settings [1]. Consequently, the traditional systems find it difficult to model spontaneous interactions or environmental reacts in intricate pictures [2]. Conversely, the present-day intelligent animation systems use AI-based learning methods that allow for autonomous and adaptable behavior creation, thus, increasing the realism and flexibility of the animated scenes.

RL is important to allow the intelligent decision-making in the field of the animated systems. In RL, a system agent acts on an environment and acquires the best behavior based on trial-and-error experiences directed by rewards and penalties [3]. The agent would overtime come up with policies that maximize cumulative rewards, yet are efficient in the way they carry them out [4]. With animation characters modeled as RL agents, it is possible to automatically learn a complex behavior, e.g., navigation, obstacle avoidance, interaction with objects, and collaborative behavior, without any explicit programming [5]. The natural, diverse and adaptive patterns of motion generated in this learning method are more natural than the scripted animation of rigid bodies. Moreover, RL allows characters to constantly change their behavior depending on the environmental changes, so the animated scenes are becoming more dynamic and lifelike.

CV in its turn is a supplement of the RL, which enables a perceptual intelligence that enables animated agents to process visual information of the environment. CV methods capture significant details in the images or video frames, allowing agents to recognize objects, approximate postures, identify scene structure, and motion tracking [6]. These features enable the agents to have ideas about the spatial associations and those environmental factors, which are pertinent in making decisions [7]. As an example, objects recognition assists characters to identify an obstacle or a target, and scene classification and tracking of motion aid world recognition and active use in animated worlds. This type of visual knowledge will allow agents to act smarter and react accordingly to alterations in the scene.

By combining RL and CV we will have a robust structure of intelligent animation systems. In this synergy, CV modules give perceptual information which analyses visual features, and the RL algorithms decide the most suitable course of action by based on learned policies [8]. The combination facilitates adaptive scene manipulation, scene-aware character interaction and environmental aware animation behavior. Consequently, this makes animated systems able to create self-adaptive, interactive and responsive scenes that are almost similar to dynamic ones in the real world [9]. These developments find significant use in the field of video games, virtual reality environments, simulations platforms, robotics training and interactive storytelling in which realism and ability to customize is highly necessary.

Fig 1 shows the graphical abstract of the research. Fig 2 depicts a combination of computer vision, reinforcement learning, adaptive optimization, and feature analysis in real time generation in animated scenes. The framework brings into focus major steps such as preprocessing of the data, extraction of visual features, intelligent decision making, and optimization of the parameters and performance of the system. The system improves the quality of animation, as well as the computing efficiency, and responsive scene creation, however, through this integrated pipeline, next-generation intelligent animation technologies are also developed.

**Fig 1:**
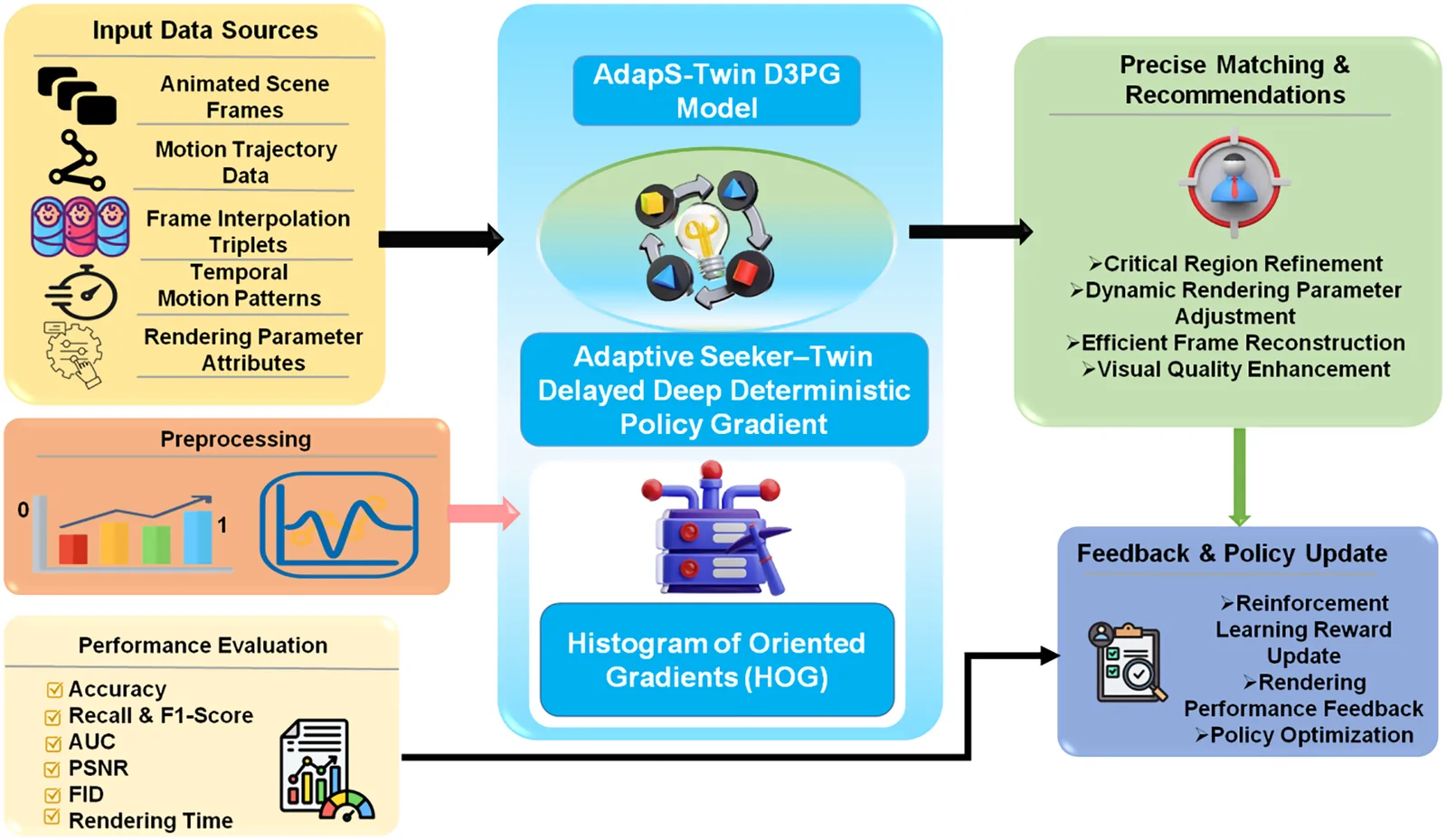
Graphical abstract.

**Fig 2:**
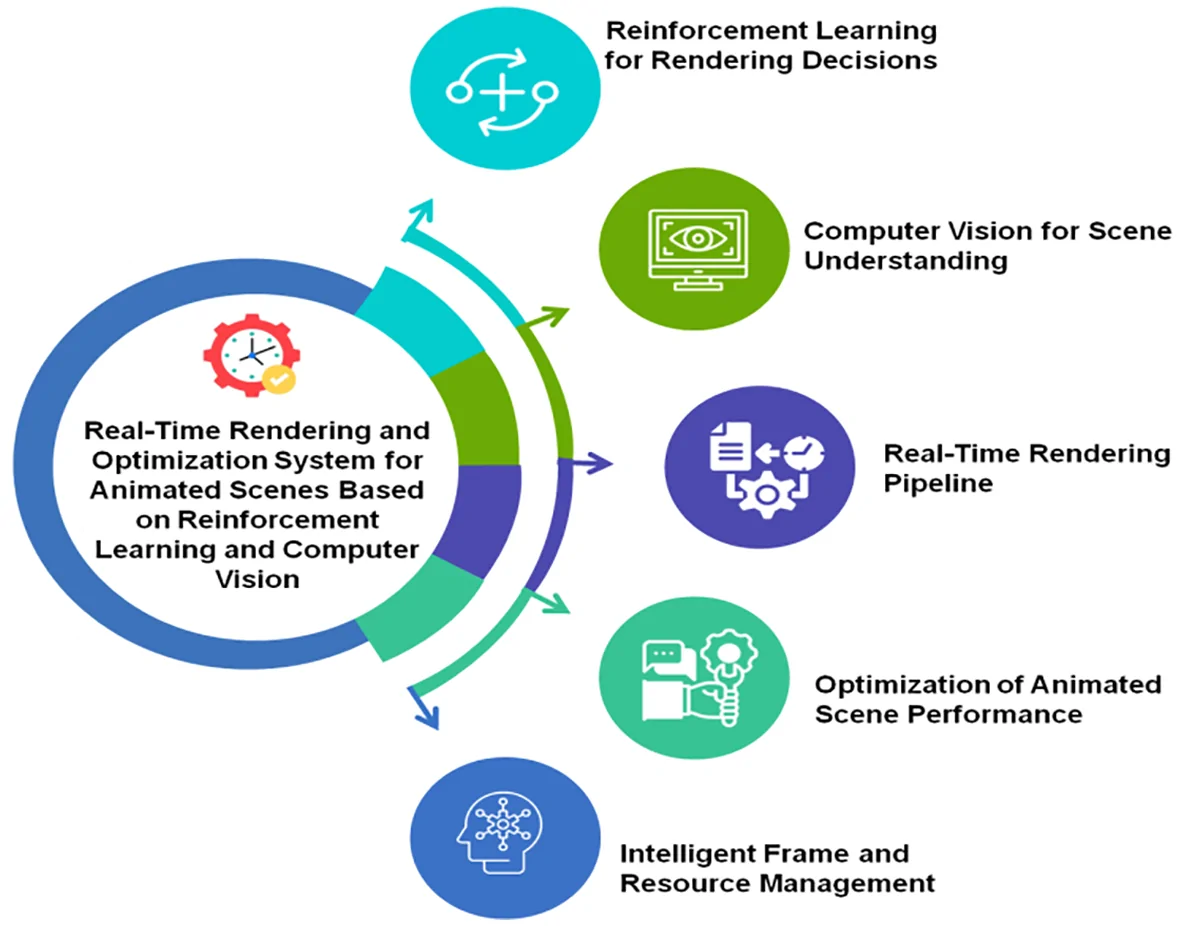
Framework of Real-Time Rendering Optimization System Using RL and CV.

Conventional systems of generating animation scenes are typically based on the techniques of Machine Learning (ML) and Deep Learning (DL) to examine motion in patterns and produce realistic visual sequences. Support Vector Machines (SVM) are one of such methods and are more often applied to the task of motion classification as they can distinguish more complex patterns in the feature space [10]. K-Nearest Neighbors (KNN) is utilized to forecast motion patterns in motion pictures characters through the recognition of similarities between samples of motion. The Hidden Markov Models (HMM) are extensively employed to model sequential data and thus allow modeling of temporal transitions in animation frames. Artificial Neural Networks (ANN) help to classify the input features to the actions to enable the motion behaviors to be generated autonomously. Convolutional Neural Networks (CNN) is utilized to capture spatial details of image frames and they are used to recognize meaningful visual elements of an animated frame [11]. To perform a temporal modeling, Recurrent Neural Networks (RNN) and Long Short-Term Memory (LSTM) networks will be applied to model sequential dependencies and regulate the frame-by-frame dynamics of animations. Also, Generative Adversarial Networks (GAN) was applied to create novel frames or textures by being trained to learn data distributions on training models.

Although effective, traditional ML and DL algorithms can be characterized by various limitations in the application to complex environments of animated scenes. Most of these methods require substantial amounts of labeled training information that in turn makes the data preparation process cumbersome and prevents the methods to be scaled to novel or unobserved situations [12]. Moreover, deep learning systems, including CNN, RNN, and LSTM, tend to be ineffective in the case of learning long-term dependence and cannot adapt as well in actual real-time rendering scenarios. When learning to generate a model, GAN-based approaches may provide unstable results, whereas classical algorithms, such as HMM and SVM, have scaling issues when using high-dimensional data of animations. Consequently, they often make it impossible to utilize such systems to autonomously utilize decision-making to optimize dynamically the animation pipeline. To address these issues, an adaptive smart framework involving CV and RL by incorporating the AdapS-Twin D3PG algorithm was created to facilitate efficient decision making, efficient control as well as enhanced rendering quality in real time animated scene settings. Fig 3 shows major innovations improving rendering efficiency, quality, and adaptive optimization.

**Fig 3:**
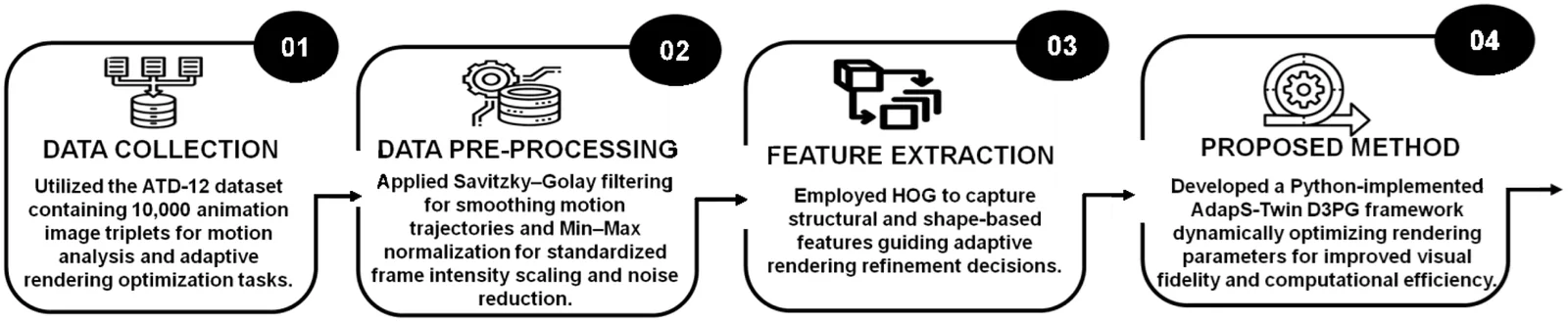
Key Contribution of RL-Based Animated Scenes in Real-Time Rendering Optimization System.

## Literature review

The smart animation special effects system through the merger of Computer-Aided Design (CAD), RL, and CV to do automated scene modeling, behavior learning, and effects generation was formulated [13]. The consequences are better realism and efficacy. Nevertheless, it is more costly to compute, model training is complicated, and it requires the presence of large datasets with annotations. The animation rendering through combination with CAD modeling and RL to improve rendering efficiency and visual quality in dynamic scenes were reviewed [14]. The findings are better real time performance, smooth scenery generation, and enhanced visual effects. It has challenges such as computational complexity, strong hardware reliance, and low dataset generalization. The smart animation with working CV, CNN, and LSTM combined with multimodal emotion recognition to improve the production of Chinese animated films was tested [15]. Findings indicate better emotional atmosphere and expressiveness of character. Limitations encompass high computational complexity, a high amount of training data and a low capacity to scale in real time.

The 3D human animation and action recognition with ML, CNN, and millimeter-wave point cloud with Lie Group modelling [16]. It had an accuracy of 97.26% and consistent virtual interaction. Disadvantages are a small self-constructed dataset, testing, and computational complexity as well as generalization issues in practice. A system of human motion recognition with two-layer LSTM network to automatically derive motion capture data time features and classify six motion patterns in real-time was tested [17]. Findings indicate that recognition accuracy has been enhanced than four DL models. Its shortcomings are dependence on sets of data, the complexity of the computations, and the complexity of extrapolating on the basis of not-seen variations of motion. The First Order Motion Model (FOMM) with an improved Convolutional Block Attention Module (CBAM) and a repainting repair block to produce strong animated characters was improved [18]. VoxCeleb1 and TaiChiHD results reveal a better peak Signal/Noise ratio (PSNR) and Structural Similarity Index Measure (SSIM). Issues are that it is computationally complex and performant in worsted under extreme pose variations.

Table 1 addresses recent contributions to the study on 3D animation, describing the methodologies, optimization, and performance metrics, and the field of application to compare the developments in reinforcement learning and GAN optimization, computer vision, and virtual reality systems.

**Table 1:** Related works of recent advances in 3D animation technique.

Ref. No	Methodology	Key Contribution	Performance Metrics	Application Domain
[19]	Duration-Sensitive Asynchronous Advantage Actor-Critic (A3C-RL)	Improved 3D animation video QoE by classifying videos based on duration and optimizing reward functions using RL	Higher QoE compared to state-of-the-art methods	3D Animation Streaming & Network Optimization
[20]	Generative Adversarial Network optimized with Particle Swarm Optimization	Enhanced virtual character animation quality through GAN hyperparameter tuning using PSO	SSIM: 0.75 → 0.88; PSNR: 24.5 → 28.7 dB; FID: 50.3 → 30.2; Reduced training &inference time	Virtual Character Animation, Gaming, VR
[21]	Deep Learning-based Facial Feature Extraction + Support Vector Machine	Real-time facial expression capture and 3D animation generation with high detection accuracy and speed	Improved face detection accuracy and real-time performance	Human–Computer Interaction & 3D Animation
[22]	Computer-Aided VR with Animation Material Ontology Library	Developed an ontology-based animation material classification system for automatic 3D scene generation	Improved interoperability, structured material composition framework	3D Scene Design & Digital Sculpture
[23]	CV based 3D Reconstruction	Applied CV for scene and character 3D reconstruction to enhance film special effects	Higher viewer interest (3D: 106 vs 25); Stronger experience perception	New Media Film & Television Special Effects

Table 2 summarizes existing AI-driven approaches for 3D animation scene generation and CAD modeling, comparing methodologies, core techniques, performance outcomes, and contributions toward improving modeling accuracy, rendering efficiency, and automation.

**Table 2:** Comprehensive studies of AI methods for animation scene modeling.

Ref. No	Methodology	Core Techniques	Key Results	Contribution to 3D Animation / CAD
[24]	Improved GAN for 3D Animation Scene Generation	CNN, Earth-Mover (EM) Distance, Least Squares Method (LSM), Label Smoothing, Deep Convolutional GAN	LSM model achieved lower loss (G: 0.3–0.5, D: 0.1–0.2); Inception score improved by 0.0297 over CNN & EM variants	Enhanced stability, reduced loss variation, and improved 3D animation image quality
[25]	CNN-based Object Recognition with CAD Modeling	Convolutional Neural Network (CNN), Boundary Extraction, CAD Model Reconstruction	Recognition accuracy >93%; high modeling precision; acceptable runtime	Automated object recognition and CAD model generation for animation scenes
[26]	Generative Modeling with Machine Vision for CAD Scene Optimization	Machine Vision, Generative Modeling, Segmentation Algorithms, Feature Fusion	High segmentation accuracy; low computational complexity; strong classification performance	Improved CAD rendering efficiency and real-time interactive animation scene design
[27]	AI-based CAD Parametric Design for Animation Scenes	DL, Machine Vision, Parameter Constraint Modeling	Lower error rates; higher accuracy than traditional methods	Automated scene element extraction and parametric animation design optimization

A real-time system of character motion generation that combines Temporal-Stylistic Latent Animator (TSLA) and Domain-Informed Animation Realignment Strategy (DIARS) to achieve realistic and flexible animation was created [28]. Findings indicate higher animation completion, style transfer and semantic editing. Disadvantages are that it is computationally complex, requires dependency on a dataset, and cannot be generalized to previously unknown movements. The image quality and real-time performance of film and television animation special effects on a DL-based optimization model were further increased [29]. Findings indicate that rendering time is shorter and quality of detail and texture is better than the physics-based and particle system techniques. There are dependence on datasets and high cost of training. Redesigned Convolutional Block Attention Model (CBAM) and a repainting image repair module to enhance animated character image generation were analyzed in the First Order Motion Model (FOMM) [30]. The findings are better Peak Signal-to-Noise Ratio (PSNR) and Structural Similarity Index (SSIM). Disadvantages are complicated calculations and loss of performance on extreme pose variation. Research explored the application of the Reinforcement Operator Learning (ROL) technique using the combination of DeepONet and Twin-Delayed Deep Deterministic Policy Gradient (TD3), testing the approach on one-dimensional Kuramoto–Sivashinsky test problem [31]. The approach showed 64.3% energy reduction in comparison with LQR and TD3, correspondingly. However, it was tested only on one benchmark problem.

### Research gaps

Despite making considerable steps forward in artificial intelligence as a 3D animation and CAD-developed rendering system, there are still a number of restrictions to their application in reality. Several of the current methods require huge amounts of computation and special hardware. This limitation limits their applicability towards working efficiently in real time settings, especially when using it in large-scale animation pipelines, or in interactive applications, as well. Moreover, many more modern models are also highly dependent on large amounts of training data that include annotations. Using such data compromises the flexibility of these systems to new scenes, novel motion patterns or unstructured environments where labeled data is limited. The other significant problem is the generalization ability of the existing AI-based animation systems. Most models will not perform very well when subjected to extreme changes in poses, high-speed motion and complicated lighting. This weakness makes them not effective in practice in the real world where the dynamics of the scenes and the environmental factors cannot be predicted. What is more, by incorporating several intelligent modules, e.g., RL, generative adversarial networks (GAN), CV, and CAD-simulation, one tends to get a more complex system. All components imply extra training needs, hyper parameter optimization issues, and inter process communication that influence overall efficiency and stability altogether. The suggested AdapS-Twin D3PG constructive addresses these shortcomings by introducing a conceptualization of adaptive rendering optimization strategy. The model is a dynamic approach to control the rendering parameters by evaluating the visual significance in the animated scenes. Areas that hold important visual information are given particular computing resources to ensure high quality in the rendering, and other insignificant areas are rendered in rough manner. Such selective allocation of resources makes unnecessary computation, as well as processing efficiency without limiting visual fidelity. Therefore, the suggested method improves scaling, reduces the computing cost and is a more efficient and powerful solution to rendering intelligent animated scenes in complex setups.

## Methodology

The methodology will start with the data collection of the ATD-12 dataset that has 10000 animation image triplets used to perform motion analysis and frame interpolation activities. Preprocessing contains use of Savitzky-Golay filter to smooth movement direction and maintain temporal dynamics, and Min-Max normalization to regularize frame intensity values. The HOG is used to extract the structural and edge-based information as part of feature extraction. The suggested AdapS-Twin D3PG RL system subsequently autonomously regulates the rendering parameters, selective refining of important areas to enhance optimum imaging and computation intervals. Fig 4 displays the pipeline from preprocessing, feature extraction, learning, optimization, to rendering.

**Fig 4:**
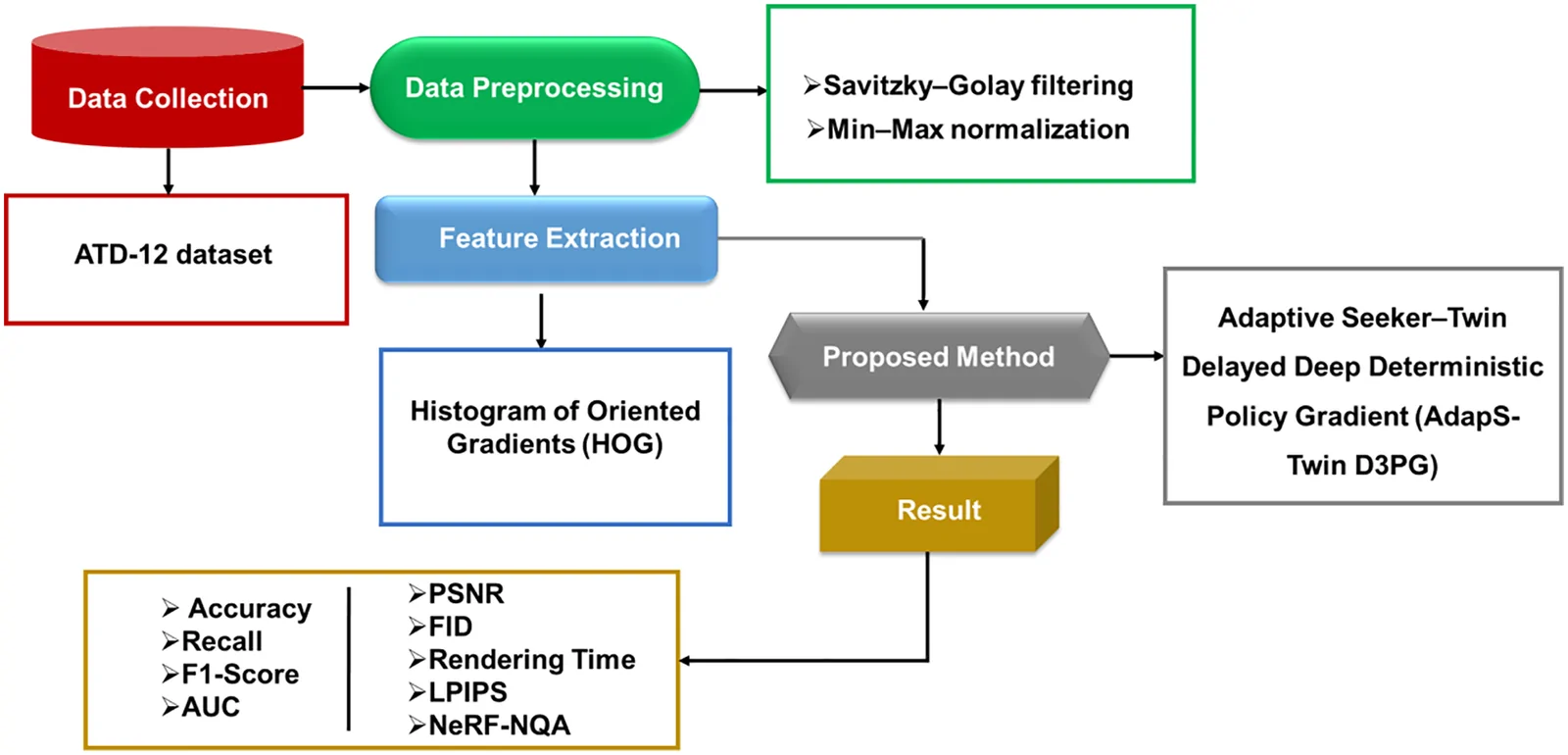
Overall workflow of real-time animated scenes using CV and RL.

### Data collection

The ATD-12 dataset available on Kaggle consists of 10,000 collected animation image triplets designed for video frame interpolation and motion analysis tasks. The dataset contains sequential animation frames that capture temporal motion dynamics between consecutive scenes. For experimental evaluation, 80% of the data is used for training and 20% for testing, ensuring balanced model development and reliable performance validation in CV research applications.

**Source:** https://www.kaggle.com/datasets/marafey/atd-12-dataset.

### Data preprocessing

Preprocessing Data Min-Max normalization is used to scale frame intensity and motion features to some common range to enhance the stability of training. The Savitzky- Golay filtering removes motion trajectories and coats the important patterns without decreasing its noises so that it can optimize the rendering process with the right pattern.

#### Savitzky-golay filtering is used to smooth motion trajectories, preserving essential temporal signal patterns.

The Savitzky-Golay filtering is used to smooth the motion tracks and frame-wise rendering measurements without violating major temporal trends and geometric characteristics. This reduces noise, stabilizes dynamic scene variations, and improves reinforcement learning–based adaptive optimization accuracy. Savitzky-Golay filter with varying order values and frame sizes. Care is taken to ensure that the order of the polynomial does not exceed the frame size, especially when the values change frequently. The polynomial order is altered from 2 to 10 and the frame size from 15 to 25 in steps of two. Over the range of values, the filtered signal is obtained and evaluated. The correlation (COR) between the filtered signal ranges in equation (1) and the original synthetic signals may be used to evaluate the trend of filtration action for different parameter values.


$$\begin{array}{c} COR=\frac{\sum_{j=\text{1}}^{m}\left(W_{j}-W)(W_{j}-W\right)}{\sqrt{\sum_{j=\text{1}}^{m}{\left(W_{j}-W\right)}^{\text{2}}{\left(W_{j}-W\right)}^{\text{2}}}} \end{array}$$ (1)

Dataset$\;W=\left\{W_{\text{1}},\ldots \ldots ,W_{m}\right\}$*,* the dataset COR is the sample mean. The term $\sum_{j=\text{1}}^{m}\left(W_{j}-W)(W_{j}-W\right)$ represents covariance, while $\sum_{j=\text{1}}^{m}{\left(W_{j}-W\right)}^{\text{2}}{\left(W_{j}-W\right)}^{\text{2}}$ represents the variance product.

#### Min-max normalization is used to scale feature values, ensuring consistent input representation.

Min–Max normalization is applied during data preprocessing to scale motion trajectories, pixel intensity values, and rendering parameters into a fixed range (0–1), ensuring numerical stability, faster convergence, balanced feature contribution, and improved learning efficiency in reinforcement learning–based real-time animated scene optimization systems. The most common method used to normalize the information is the min-max normalization process. This method adjusts the absolute minimal and absolute maximal values of the input data component to 0 and 1, respectively, and converts all other integers to decimals with values between 0 and 1. Equation (2) determines the normalized value of each input element.


$$\begin{array}{c} X_{j}^{\prime }=NeX_{min}+\left(NeX_{max}-NeX_{min}\right)*\left(\frac{X_{j}-X_{min}}{X_{max}-X_{min}}\right) \end{array}$$ (2)

Where ${\text{X}}_{\text{min}},{\text{X}}_{\text{max},\;}$to$\text{ Ne}{\text{X}}_{\text{max}}-\text{Ne}{\text{X}}_{\text{min}}$ statistics were proportionally modified. This method preserves the ${\text{X}}_{\text{j}}^{\prime }$ integrity of all data-value relationships, $\left(\frac{{\text{X}}_{\text{j}}-{\text{X}}_{\text{min}}}{{\text{X}}_{\text{max}}-{\text{X}}_{\text{min}}}\right)$making it a better choice. Bias couldn’t be introduced into the data.

#### Feature extraction using HOG is used to capture edge and structural visual features.

The use of HOG is to identify strong structural and motion-related characteristics of animated frames through the capturing of edge orientations and gradient distributions. These attributes facilitate effective scene perception, detection of object boundaries as well as dynamic region, and reinforcement learning-based rear optimization of adaptive rendering dynamically in real time. The vertical and horizontal gradient of the input image are computed with equations (3) and (4). The horizontal gradient ${\text{H}}_{\text{w}}$ is obtained by convolving the preprocessed image ${\text{J}}_{\text{e}}$ with the kernel [−1,0,1] to capture intensity changes across width, while the vertical gradient ${\text{H}}_{\text{z}}$ uses the transposed kernel ${\left[-\text{1},\text{0},\text{1}\right]}^{\text{S}}$ to detect height-wise variations.


$$\begin{array}{c} H_{w}=J_{e}*[-\text{1},\text{0},\text{1}] \end{array}$$ (3)

$$\begin{array}{c} H_{z}=J_{e}{*[-\text{1},\text{0},\text{1}]}^{S} \end{array}$$ (4)

Equations (5) and (6) are then applied to the gradients thus obtained to determine their magnitude and angular orientations. The gradient magnitude n(w,z) combines horizontal ${\text{h}}_{\text{u}}^{\text{2}}(\text{w},\text{z})$ and vertical ${\text{h}}_{\text{g}}^{\text{2}}(\text{w},\text{z})$ gradients to measure local motion strength at pixel θ(w,z). The gradient orientation ${\text{tan}}^{-\text{1}}$, computed using ${\text{h}}_{\text{v}}(\text{w},\text{z})$, and ${\text{h}}_{\text{g}}(\text{w},\text{z})$, indicates movement direction essential for modeling dance posture transitions, ${tan}^{-\text{1}}$is computes gradient orientation using inverse tangent.


$$\begin{array}{c} n(w,z)=\sqrt{h_{u}^{\text{2}}(w,z)+h_{g}^{\text{2}}(w,z)} \end{array}$$ (5)

$$\begin{array}{c} \theta (w,z)={tan}^{-\text{1}}\left(\frac{h_{v}(w,z)}{h_{g}(w,z)}\right) \end{array}$$ (6)

The image was separated into cells by it. A block made up of different cells is created, and the blocks are convolved to create features. Let ${\text{S}}_{{\text{hog}}_{\text{fs}}}$s stand for the total quantity of features that were calculated with the HOG descriptor. Blocks per image are shown by ${\text{A}}_{\text{img}}$, the block size is shown by ${\text{A}}_{\text{t}}$, and the bins used is indicated by ${\text{M}}_{\text{a}}$. Equation (7), which is mentioned below, can be used to calculate the total number of features in the subsequent formula.

Computer vision methods use HOG to extract features of visual importance from the animation frames. Edge orientation, gradient magnitude, object boundaries, texture variations, and motion features of the video frame are extracted. Perceptual importance maps are created based on the extracted features for guiding the reinforcement learning algorithm. Regions that have visual importance are assigned high priority during rendering while other regions that have less importance can be rendered with less computation.


$$\begin{array}{c} S_{{hog}_{fs}}=A_{img}*A_{t}*M_{a} \end{array}$$ (7)

HOG was chosen because optimization through rendering mainly demands better structural edges, contours, and motion gradients information instead of higher dimensional semantics. HOG is less computationally complex and deterministic as opposed to deep visual embedding, thus being more favorable for optimization in real-time rendering. AdapS can be thought of as an augmentation to Twin D3PG that explores the space of rendering parameters before optimization of the policy, while Twin D3PG ensures stable optimization in continuous control tasks through use of twin critics and delayed policy updating.

#### AdapS-twin D3PGused to optimize rendering parameters adaptively for efficiency.

The hybrid AdapS-Twin D3PG framework is designed to enhance real-time rendering and optimization in complex animated scenes by integrating RL with computer vision. This model builds upon the principles of Twin D3PG with an adaptive seeker module that analyses dynamically scene complexity, object motion, lighting variation and camera transformations. The convolutional networks are used to derive visual features that help the agent predict rendering importance maps to allocate resources selectively. AdapS has four feedback-based control loops that continuously check frame-level statistics like frame rate, latency, and visual distortion. Depending on the policy gained, the system varies in shading resolution, rates of texture sampling, and geometric detail real time so as to resource both the level of computational efficiency and visual fidelity. AdapS-Twin D3PG provides faster convergence, better stability, and consistent quality rendering behavior at high levels with a variety of animated scene settings by using perceptual awareness encouragement with CV and stable continuous control with deep reinforcement learning. Algorithm 1 shows the procedure of the AdapS-Twin D3PG method.

The Adaptive Rendering Policy for Rendering in Animated Scenes using AdapS-Twin D3PG reinforcement learning that can enhance rendering performance by adaptively adjusting the rendering process through interaction with the animated scenes. This involves the RL agent understanding scene complexity, dynamics of motion, changes in lighting, and camera dynamics and then adapting the rendering policy by adjusting parameters such as shading granularity, texture sampling rate, and geometric detail. Resources can be allocated selectively to areas that need more attention while ignoring other less important areas, resulting in enhanced visuals without waste of computing resources.

**Algorithm 1: AdapS-Twin D3PG**

Load ATD-12 dataset D (10,000 animation frame triplets).

Split dataset into 80% training and 20% testing.

For each frame J in D do

Apply Savitzky–Golay filtering to smooth motion trajectories.

Apply Min–Max normalization:


$$\begin{array}{c} X^{\prime }=\frac{X-X_{\text{min}}}{X_{\text{max}}-X_{\text{min}}} \end{array}$$

End for.

For each preprocessed frame Je do

Compute horizontal gradient:


$$\begin{array}{c} H_{w}=J_{e}*\left[-,\text{1},\text{0}\text{1}\right] \end{array}$$

Compute vertical gradient:


$$\begin{array}{c} H_{z}=J_{e}*{\left[-,\text{1},\text{0}\text{1}\right]}^{T} \end{array}$$

Compute magnitude n(w,z)and orientation θ(w,z).

Form HOG histograms and construct feature vector $S_{hog}$.

End for.

Initialize Actor network $\pi_{\phi }$.

Initialize Twin Critic networks $Q_{\theta_{\text{1}}}$ and $Q_{\theta_{\text{2}}}$.

Initialize target networks $\phi^{\prime },\theta_{\text{1}}^{\prime },\theta_{\text{2}}^{\prime }$.

Initialize replay buffer R.

Initialize AdapS seeker population with bounds $\left(\mu_{\text{min}},\mu_{\text{max}}\right)$.

For episode =1 to N do

Initialize state t using HOG features.

For each step until terminal state do

Compute adaptive inertia weight:


$$\begin{array}{c} \omega =\mu_{\text{min}}+\left(\mu_{\text{max}}-\mu_{\text{min}}\right)\times rand+\text{0}.\text{2}\times randn \end{array}$$

Update seeker positions using ω.

Apply boundary reflection strategy.

Evaluate fitness E.

Select optimized seeker action component $b_{adaps}$.

Generate exploration noise:


$$\begin{array}{c} \varepsilon \sim clip\left(N,\left(\text{0},\sigma \right),-dd\right) \end{array}$$

Compute reinforcement learning action:


$$\begin{array}{c} b=\pi_{\phi }(t)+\varepsilon \end{array}$$

Execute combined action $\left(b_{adaps}+b\right)$.

Observe reward qand next state $t^{\prime }$.

Store $\left(t,b,q,t^{\prime }\right)$in replay buffer R.

Sample mini-batch from R.

Compute target value:


$$\begin{array}{c} z=q+\gamma \times \text{min}\left(Q_{\theta_{\text{1}}}^{\prime },Q_{\theta_{\text{2}}}^{\prime }\right) \end{array}$$

Update $\theta_{\text{1}}$ and $\theta_{\text{2}}$ by minimizing MSE loss.

If step mod 2=0then

Update actor parameters ϕ using policy gradient.

Soft update target networks:

$\theta^{\prime }\leftarrow \tau \theta +(\text{1}-\tau )\theta^{\prime }$ $\phi^{\prime }\leftarrow \tau \phi +(\text{1}-\tau )\phi^{\prime }$

End if.

Set $t=t^{\prime }$.

End for.

End for.

Return optimized rendering policy $\pi_{\phi }$.

End

#### Twin D3PG used to reduce value overestimation, improving reinforcement learning stability.

Twin D3PG is a state-of-the-art, RL-based optimizing system developed to render in real-time in an animated scene. It expands upon DDPG by incorporating twin critic networks, delayed policy updates and target smoothing as a way of reducing the overestimation bias and enhancing stability. The Twin D3PG, a part of this system, is an adaptive system that determines rendering parameters based on CV-based analysis of the scene, provides an optimal frame rate, visual fidelity, and computational efficiency in dynamic (high-complexity) scenes. Overestimation of the value function harms the operation of the Q-learning algorithm and, in continued cases during training, the updating of policies. In response to the problem, two-value networks derived techniques such as Double Q-Learning and Double DQN were developed. In addition, in Twin D3PG, a variety of methods are applied to minimize the overvaluation of the value function in Deep Deterministic Policy Gradient (DDPG).

Two techniques of Twin D3PG algorithm that are discussed in the text are Clipped Double-Q Training policy and Delayed Policy Updates. Clipped double-Q learning aims at learning two Q-functions, i.e., two actor networks, two critic networks and two Bellman equations. The method is referred to as delayed policy updates, which is switching between policy and target populations not as often as the Q-function to minimize variations in value estimates, a more consistent set of values, and minimizing errors before updating the policy network.

Twin D3PG explores the space by a process known as Target Policy Smoothing, which entails the introduction of noise to the policy to prevent determinacy. Twin D3PG uses clipped random noise to the goal action while training off-policy, in contrast to on-policy approaches that might not explore enough. The z denotes the target Q-value for critic update; q is immediate reward; γ the discount factor; $R_{x}$ critic evaluation at next state $t^{\prime }$; $\mu_{\theta }\left(t^{\prime }\right)$ actor-generated action;ε ~ clip is clipped noise added for stable policy updates,ε denotes noise added to the target policy for exploration and overfitting prevention. M (0, σ)is Gaussian noise with mean 0 and standard deviation σ, clipped between −d and d for stability in equations (8)–(9).


$$\begin{array}{c} z=q+\gamma R_{x}(t^{\prime },\;\mu_{\theta }\left(t^{\prime }\right)+\varepsilon ) \end{array}$$ (8)

$$\begin{array}{c} \varepsilon \;\sim \;clip\;(M\;(\text{0},\;\sigma ),\;-d,\;d) \end{array}$$ (9)

To ascertain the parameters of Twin D3PG, the parameters of the two critical models are obtained by back propagating the critic loss. Equation (10), represents the parameters of the $j^{th}$ critic network updated during training. M denotes the minibatch size sampled from the replay buffer, ${min}_{\theta_{j}}$ is selects minimum critic loss for stability. z is the target Q-value. $Q_{\theta_{j}}(t,b)$ represents the predicted Q-value for state t and action b.


$$\begin{array}{c} \theta_{j}\;\leftarrow \;{min}_{\theta_{j}}M^{-\text{1}}\sum_{j=\text{1}}^{M}(z-Q_{\theta_{j}}({t,b))}^{\text{2}} \end{array}$$ (10)

$$\begin{array}{c} \nabla_{\varphi }I(\varphi )=M^{-\text{1}}{\sum \nabla_{b}Q_{\theta \text{1}}(t,b)|}_{b=\pi_{\varphi }(t))}\nabla_{\varphi }\pi_{\varphi }(t) \end{array}$$ (11)

Where φ denotes actor network parameters, I(φ) the objective maximizing expected reward, $M^{-\text{1}}$ minibatch size, $Q_{\theta \text{1}}(t,b)$ critic Q-value estimate, $\pi_{\varphi }(t)$ actor policy producing action b, while $\nabla_{b}$ and $\nabla_{\varphi }$ guide actor updates in equation (11). The target networks are using equations (12) – (13), where$\theta_{j}^{\prime }$ and $\varphi_{j}^{\prime }$ denote target critic and target actor parameters, while $\theta_{j}^{\prime }$ and $\varphi_{j}^{\prime }$ represent current network parameters. τis the soft update coefficient controlling update rate, and (1−τ) preserves previous target values, ensuring gradual parameter updates that stabilize learning and prevent divergence during reinforcement learning training. Fig 5 displays a flowchart showing the actor, twin critics, replay buffer, and policy updates.


$$\begin{array}{c} \theta_{j}^{\prime }\;\leftarrow \;{\tau \theta }_{j}+(\text{1}-\tau )\theta_{j}^{\prime } \end{array}$$ (12)

$$\begin{array}{c} \varphi_{j}^{\prime }\;\leftarrow {\tau \varphi }_{j}+(\text{1}-\tau )\varphi_{j}^{\prime } \end{array}$$ (13)

**Fig 5:**
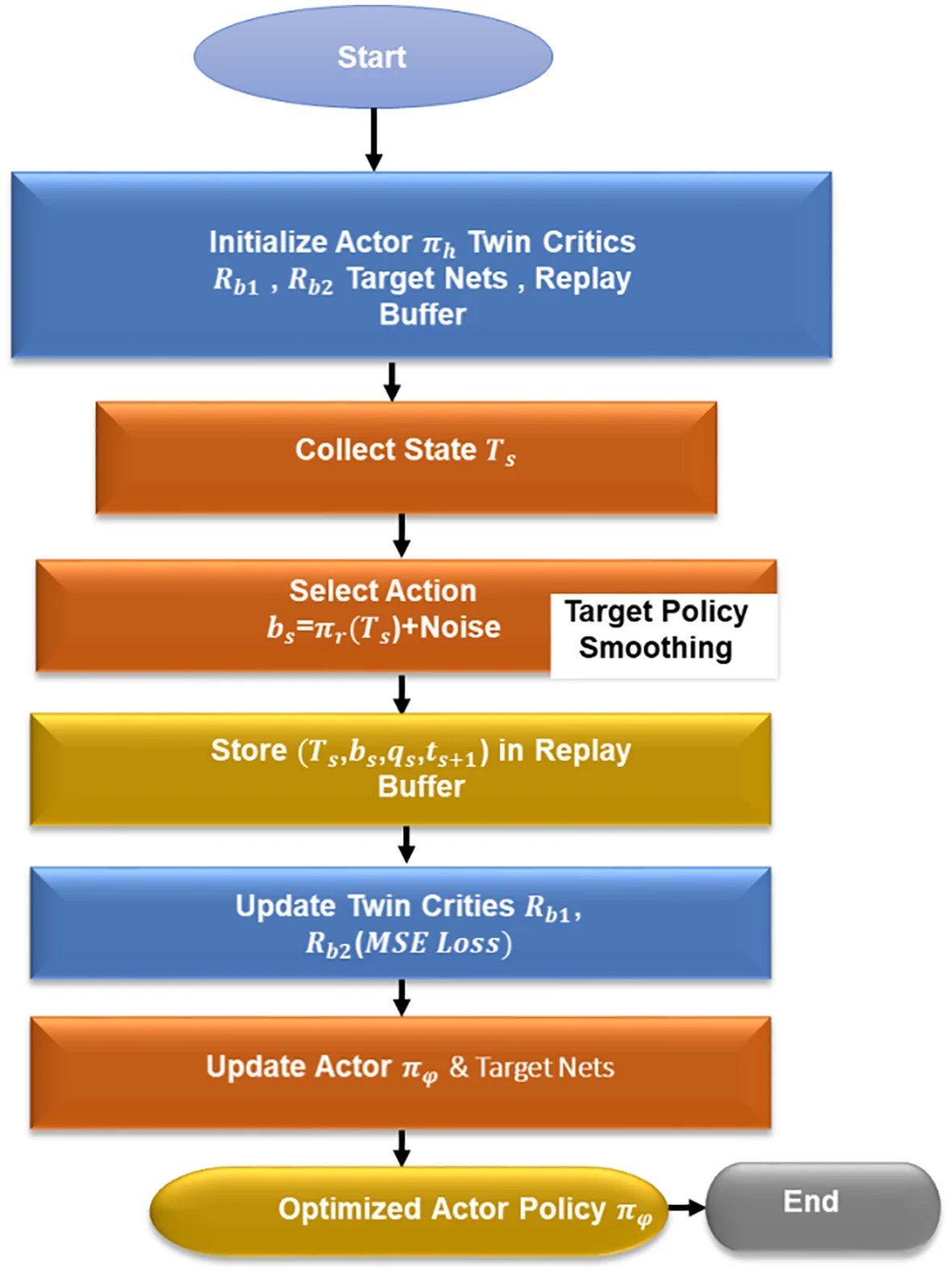
Twin D3PG RL Framework for Efficient Policy Optimization and Training.

#### AdapS used to locate important regions for adaptive rendering refinement.

AdapS is a reinforcement learning–driven optimization module designed for real-time rendering in animated scenes. Integrated with CV and RL techniques, AdapS dynamically adjusts rendering parameters based on scene complexity, motion dynamics, and resource availability. By leveraging adaptive policy learning, it balances visual fidelity and frame rate performance. The system improves computational efficiency, reduces latency, and ensures stable, high-quality output in dynamic and large-scale animated environments. Similar to other clever algorithms is the seeker optimization algorithm. Continuous iteration may cause the search process to stagnate, and in the later stages, a significant number of search participants may locally aggregate, finally leading to algorithm convergence. The outcome falls into the regional optimum with ease. As a result, the algorithm’s convergence speed and accuracy must be increased, and the issue of a lack of population variety in the algorithm’s latter stages must be resolved.

Random dynamic change in the weight of inertia: The gradient descent approach is used by the conventional SOA to adjust the inertia weight iteration. In a gradient descent approach, the learning rate is a fixed value that is often determined by experience. The learning rate value’s magnitude influences the search individual positioning update rate, which in turn influences the algorithm’s overall pace and causes it to converge to the globally optimal solution. Therefore, the randomly produced inertia weight value may be rather modest when employing the random inertia weight mutation technique if the individual is near the ideal location in the initial state. This can speed up the algorithm’s overall convergence. The gradient descent method’s inability to converge to the optimal point due to a linear decline in the weight value can be solved by using the random inertia weight mutation procedure in equation (14).


$$\begin{array}{c} \omega =\mu_{min}+\left(\mu_{max}-\mu_{min}\right).rand(\text{0},\text{1})+\text{0}.\text{2}.M(\text{0},\text{1}) \end{array}$$ (14)

In this equation, ω denotes the adaptive seeker weight controlling exploration. $\mu_{min}$ and $\mu_{max}$ represent the minimum and maximum bounds of the search parameter. rand(0,1) generates a uniform random value between 0  and 1, while 0.2.M(0,1) adds Gaussian noise with mean 0 and variance 1 to enhance randomness.

Strategy for boundary reflection: The person’s position will surpass the available space during the migration procedure. A lot of seekers may congregate at the boundary as a result of the conventional SOA’s handling of the boundary, which fixes the seekers beyond the border to the boundary. Setting boundary settings for conventional SOA algorithm processors will have an effect on population variety since they fix a high number of seekers outside the boundary region on the boundary. Inspired by reverse learning, an enhanced boundary projection strategy is obtained, and seekers beyond the upper and lower limits are captured and transferred into the population’s boundary in accordance with Equation (15). This avoids the flaws of the standard SOA, boosts population diversity, and somewhat improves the algorithm’s convergence accuracy.


$$\begin{array}{c} \left\{ \begin{array}{c} swarm\left(j,\;e\;ind\left(swarm(j,\;:)>{pop}_{max}\right)\right)={pop}_{max}-l_{\text{1}}.{pop}_{max}\;(l_{\text{1}}\geq \text{0}.\text{5})\\ swarm(j,\;e\;ind(swarm(j,\;:)\;>{pop}_{max}))\;=\;(l_{\text{1}}<\text{0}.\text{5})\\ \begin{array}{c} swarm\left(j,\;e\;ind\left(swarm(j,\;:)<{pop}_{min}\right)\right)={pop}_{min}+l_{\text{2}}.{pop}_{max}(l_{\text{2}}\geq \text{0}.\text{5})\\ swarm\left(j,\;e\;ind\left(swarm(j,\;:)<{pop}_{min}\right)\right)={pop}_{min}\;(l_{\text{2}}<\text{0}.\text{5}) \end{array} \end{array}\right. \end{array}$$ (15)

Here, $l_{\text{1}}=rand()$and$l_{\text{2}}=rand()$ are some limitations on the rand() random number produced by $j-th\;particle;{pop}_{max}\;and\;{pop}_{min}$ are the upper and lower bounds, respectively, and swarm(j, :) is the update location of the j−th particle.Where, ${pop}_{max}$ denotes the maximum population limit, $l_{\text{1}}$ is the adaptive control factor $l_{\text{1}}\geq \text{0}.\text{5}$, swarm(j, :) represents the j-th swarm solution vector, and $e\;ind\left(swarm(j,\;:)>{pop}_{max}\right)$ identifies elements exceeding ${pop}_{max}$, which are adjusted to maintain population stability.

Enhancement of the fitness purpose: Let o be the number of seekers in the population, and let each particle’s position vector consist of three PID controller control parameters, or the individual position vector’s dimension C = 3. A matrix of $T_{\text{1}}\times C$ can then be used to represent the population. The SOA algorithm only uses the value of fitness to assess the benefits and drawbacks of the individual or solution during the search and evolution process. This value is then used to update the individual’s position going forward, allowing the initial solution to progressively evolve to the optimal solution. Where E represents the total adaptive energy or cost. $\omega_{\text{1}}$, $\omega_{\text{2}}$, $\omega_{\text{3}}$ are weighting coefficients controlling contributions of different terms. f(s) denotes force or feedback signal, $v^{\text{2}}(s)$ velocity, λ a scaling factor, e(t) the error signal, and cs indicates integration over relevant state, applied only when f(s) < 0 in equation (16). Fig 6 shows the AdapS algorithm dynamically updates seeker positions, optimizing rendering parameters efficiently.


$$\begin{array}{c} E=\int_{\text{0}}^{\infty }(\omega_{\text{1}}\left|f(s)\right|+\frac{\omega_{\text{2}}v^{\text{2}}(s)}{\lambda |e(t)|}+\omega_{\text{3}}|f(s)|)cs\;,\;i\;f\;f(s)\;<\;\text{0} \end{array}$$ (16)

**Fig 6:**
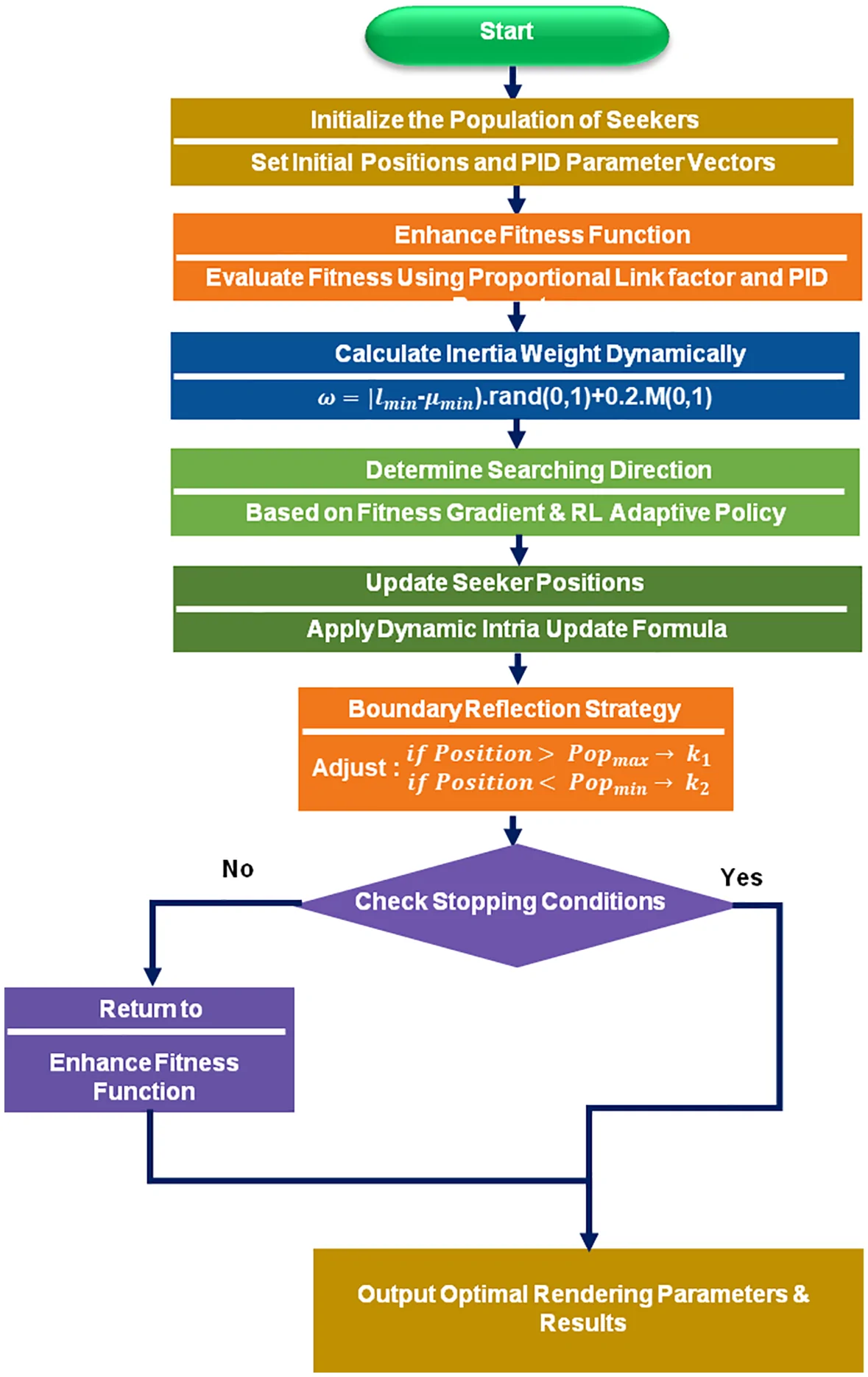
AdapS Algorithm Flowchart for Rendering Parameter Optimization.

State representation describes the environmental information that the reinforcement learning algorithm uses when observing the environment for rendering purposes at each rendering step. The purpose of the state is to give a complete depiction of the animated scene currently being seen so that the decision of rendering can be made in an intelligent manner. The state includes features of the images obtained using the HOG technique together with characteristics of the scene and the usage of hardware. computational resources. $S_{t}=\left\{H_{hog},M,L,C,F,PSU_{GPU}\right\}$ Wherein, $H_{hog}$ denotes the HOG feature vector representing structural edge information, M is the motion magnitude of animated objects, L represents lighting variation, C denotes scene complexity, FPS is the current rendering frame rate, and $U_{GPU}$ represents GPU utilization. The objective of the state representation is to accurately characterize the rendering environment so that the reinforcement learning agent can optimize rendering quality while minimizing computational cost.

The action space is where the decisions for rendering are made continuously by the reinforcement learning agent at each step of interaction with the environment. Its purpose is to adapt the rendering settings based on the properties of the scene rather than set the rendering settings statically. The continuous action space allows flexible adaptation for various animated scenes without any computational expense. The action vector is defined as $A_{t}=\left\{R_{s},T_{s},L,O,D,S_{q}G_{t}P_{r}\right\}$, wherein, $R_{s}$ denotes shading resolution, $T_{s}$ represents texture sampling rate, LOD is the level-of-detail parameter, $S_{q}$denotes shadow quality, $G_{t}$ represents geometry tessellation level, and $P_{r}$ is the rendering priority threshold used for adaptive resource allocation. The objective of the action space is to continuously optimize rendering parameters to maximize visual fidelity while reducing rendering latency and computational resource consumption.

The reward function analyzes the quality of each rendering choice and gives feedback to help learn the policies. The function aims to promote efficient rendering policies that not only increase the visual quality but also decrease the computation time without increasing the cost of rendering or producing visual artifacts. reward is formulated as $R_{t}=\alpha PSNR+\beta FPS-\gamma T-\delta E$*,* PSNR represents rendering quality, FPS denotes the achieved frame rate, T is rendering time, E represents rendering error measured using perceptual metrics such as FID or LPIPS, and α,β,γ,δ are weighting coefficients controlling the contribution of each objective. The objective of the reward function is to maximize rendering quality and rendering speed while minimizing rendering time and perceptual reconstruction errors during reinforcement learning.

Rendering parameter optimization algorithm explains the manner in which the reinforcement learning approach refines its rendering strategy by interacting continuously with the rendering environment. The goal of this algorithm is to generate a rendering strategy that adapts to the importance of the scenes being rendered while ensuring consistent performance in terms of convergence and rendering performance.

#### Ethical approval.

This research uses a publicly available dataset (https://www.kaggle.com/datasets/marafey/atd-12-dataset) that contains animated scene data without any personally identifiable information. Since no human participants, animals, or confidential personal data were involved, ethical approval was not required.

## Result

The Python-implemented model is evaluated against existing methods across multiple datasets to analyze rendering efficiency, feature extraction performance, and adaptive optimization capability. Table 3 summarizes the computational environment, software tools, and hardware configuration used to implement and evaluate the Python-based RL framework for real-time animated scene rendering.

**Table 3:** Experimental setup configuration for Python-based RL animated scene rendering framework.

Component	Configuration / Tools Used
Programming Language	Python 3.9
Development Environment	Jupyter Notebook / PyCharm
Operating System	Windows 10 / Ubuntu 20.04
Processor	Intel Core i7 / AMD Ryzen 7
GPU	NVIDIA RTX 3060 / CUDA enabled
RAM	16 GB
DL Framework	TensorFlow / PyTorch

### Hyperparameters of AdapS–Twin D3PG framework

Table 4 summarizes all key hyperparameters for AdapS–Twin D3PG, including RL learning rates, AdapS inertia bounds, preprocessing parameters, and HOG feature extraction settings, guiding stable and efficient training.

**Table 4:** AdapS–Twin D3PG hyperparameter settings and values.

Category	Hyperparameter	Value / Range
Reinforcement Learning	Discount factor (γ)	0.99
	Actor learning rate (α_a)	0.0001
	Critic learning rate (α_c)	0.001
	Replay buffer size (M)	1,000,000
	Target network update rate (τ)	0.005
	Exploration noise (σ)	0.2
	Noise clip (d)	0.5
AdapS Seeker	Population size	50
	Inertia weight min (μ_min)	0.4
	Inertia weight max (μ_max)	0.9
	Fitness function coefficient (λ)	0.5
	Max iterations per episode	20
Twin D3PG Specific	Delayed actor update frequency	2 steps
	Clipped Double-Q	Enabled

### Intelligent rendering optimization framework performance and feature analysis results

Fig 7 represents the classification performance of proposed intelligent rendering optimization framework that employs in discriminating between two rendering states. The diagonal figures show the instances of the accurate predictions by the model as 161 examples of the class 0 and 165 examples of the class 1 were correctly predicted by the model. These large highs indicate great predictive potential and consistent learning of the reinforcement learning based decision module. The misclassification is shown by the off-diagonal elements and there are 36 samples that were of class 0 but will be classified as class 1 and there are 38 samples that will be of class 1 but will be predicted to be of class 0. The number of errors that are relatively low in comparison with the correct prediction is evidence of the effective feature representation as well as the decision boundaries learnt during training. This experiment indicates that a combination of computer vision attributes, combined with reinforcement learning policy enables the system to be able to distinguish the level of importance in rendering successfully. The model is therefore able to make precise judgments on which parts of the animated scenes should have greater priority during rendering to enhance the computational efficiency and maintain the appearance quality of perceptual visual quality.

**Fig 7:**
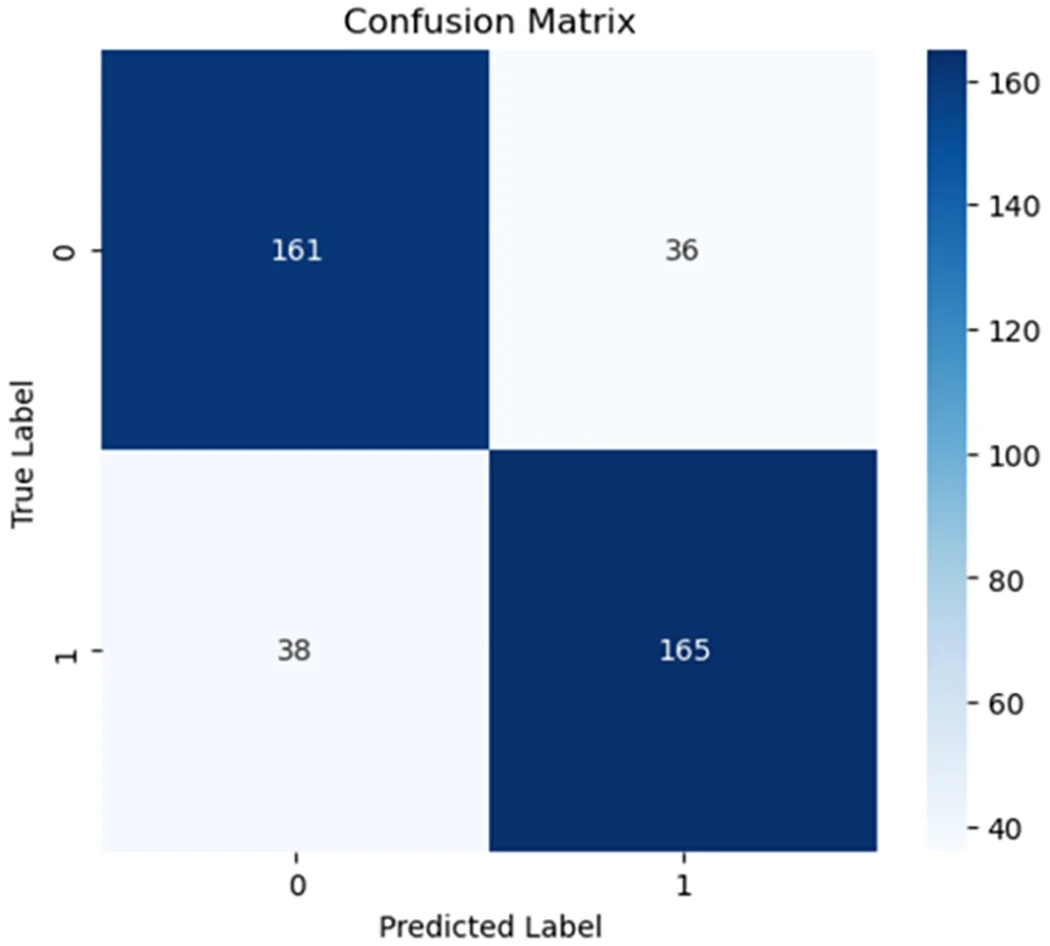
Classification performance of reinforcement learning based rendering decision model.

Fig 8 (a), shows the ROC curve, which demonstrates the performance of the reinforcement learning-based render optimum model developed to suit animated scenes. The curve is drawn to show the False Positive rate and the rates of True Positive rates at different decision levels. The model has a value of 0.901 in the area under the curve (AUC) which shows that it has the ability to determine a good rendering action and those not being the best. The curve is significantly above the diagonal Scher-line which depicts random prediction and there is considerable discrimination. The sharp increase of the first part of the curve indicates that the model is effective in recognizing a lot of optimal rendering choices at a relatively low false positive value, which proves the relevance of the reinforcement learning alongside computer vision features to the optimization of adaptive rendering. Fig 8 (b) presents the Precision -Recall curve which measures the accuracy against the ability to recall. The model has an Average Precision (AP) of 0.909, which means a high predictive reliability. At lower levels of recall, precision is extremely high and it decays slowly with increases in recall, with overall high performance being maintained.

**Fig 8:**
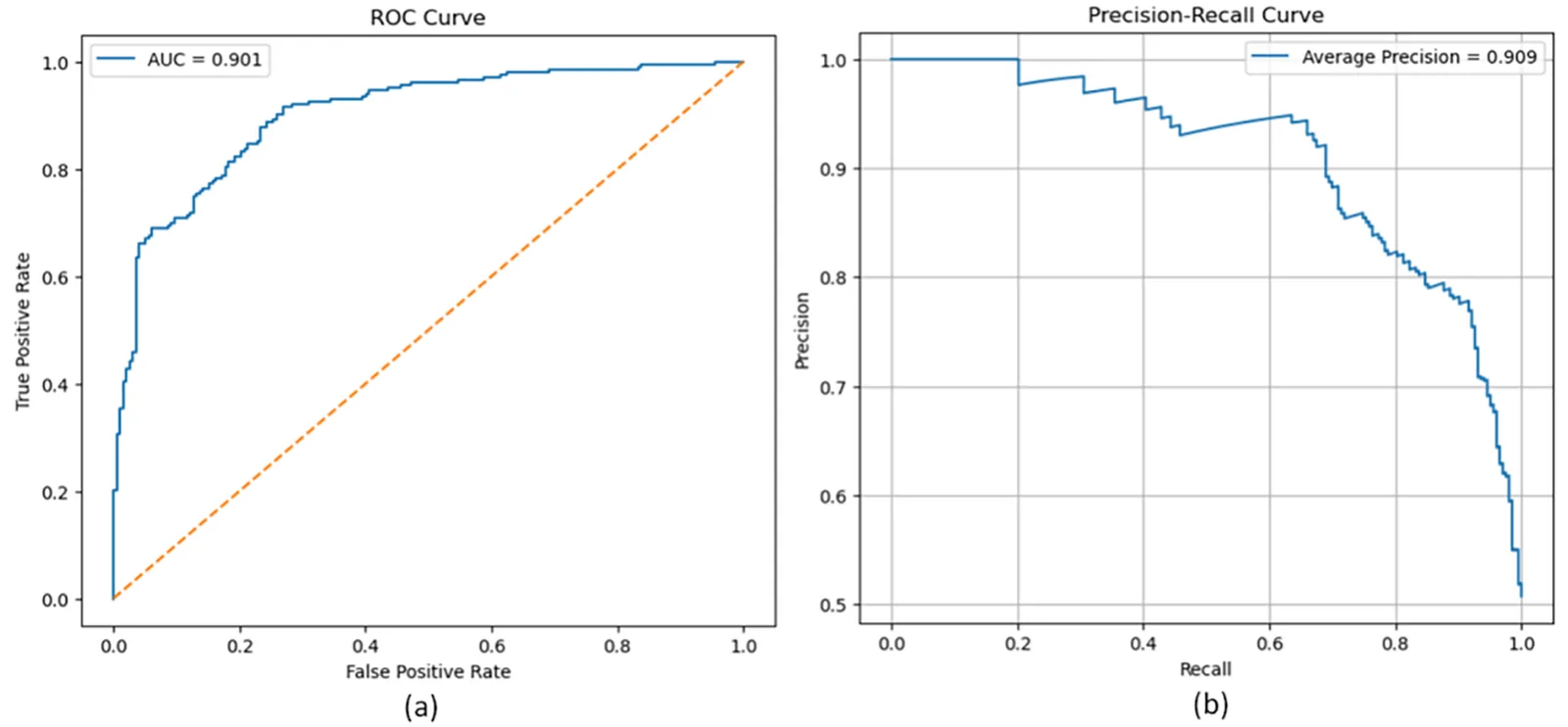
Visualization of (a) ROC and (b) Precision–Recall curves showing reinforcement learning rendering optimization performance.

Fig 9 is a statistical description of the velocity of motion retrieved as animated scene sequences in the training of a model. Horizontal axis is the velocity magnitude and vertical axis is the frequency of occurrence at sampled frames. The distribution indicates that the bulk of the motion velocities are between the range of about 2.0–3.5 units indicating that standard range of motion in animated scenes is moderate range. The low values of velocity are associated with relatively stagnant or slow-moving parts, and the high values of velocity are associated with fast movement of objects or the transition of scenes. The bell-shaped distribution implies that the motions exhibited are of near-normal form, which is positive to learn stable feature representations. Through these dynamics of motion, the reinforcement learning agent can learn to have more insight into temporal changes in the animated scenes. This data is used to make adaptive rendering decisions such that the system devotes more processing power to the more dynamic regions and less to the more stable regions so that the system is able to sustain smooth visual operation and allocation of rendering effort at the same time.

**Fig 9:**
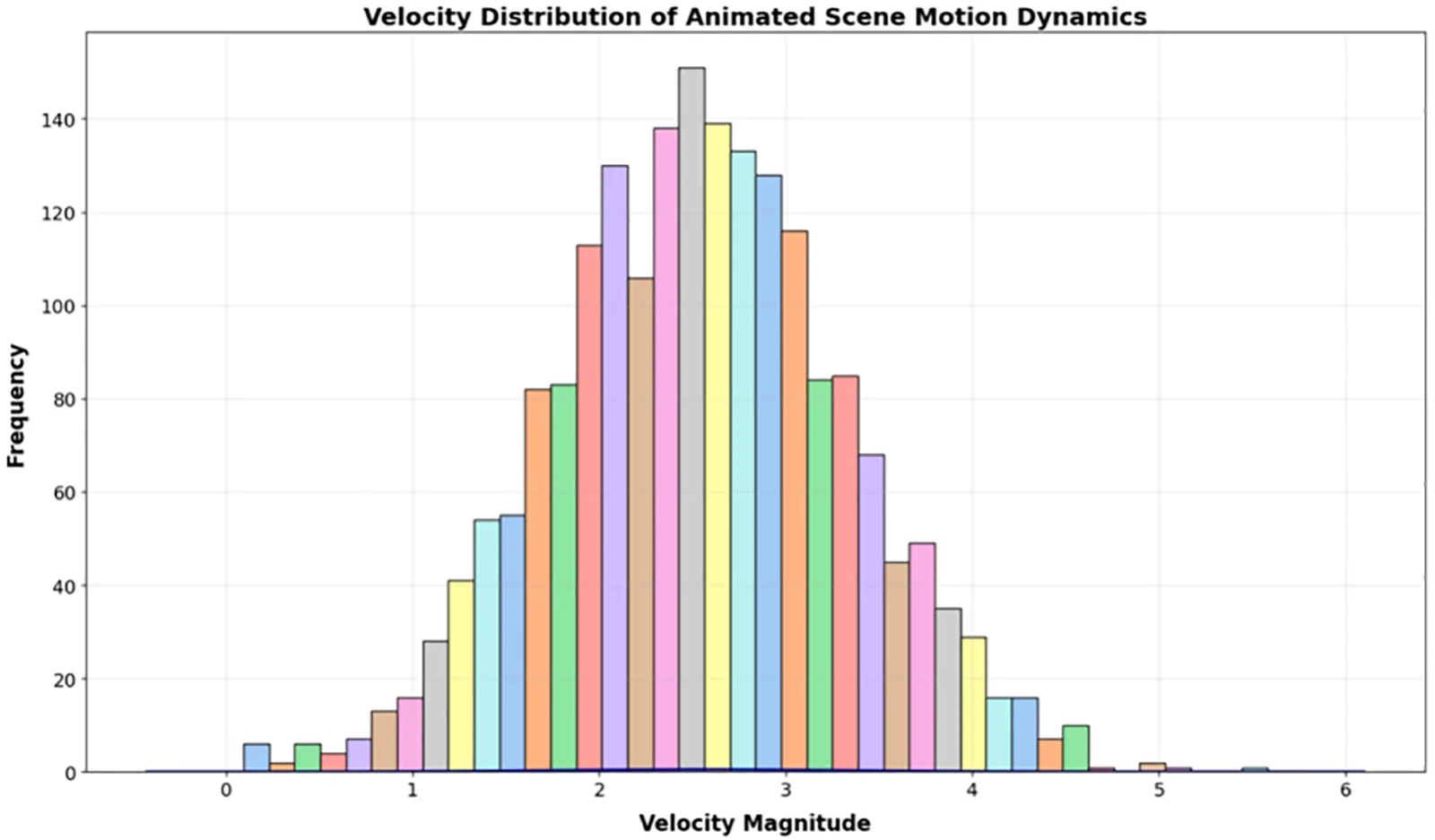
Distribution of motion velocity in animated scene dynamics.

Fig 10 shows the evolution of the performance of learning during the training steps of the intelligent rendering optimization structure. The findings show that the accuracy of the training rises drastically at the onset of the epochs which are the initial epochs to take place, which indicates that the model is able to acquire the meaningful representations of the input features fast. The accuracy then steadily levels off at the maximum value and this indicates that the learning process is converged. Simultaneously, the loss on training is steadily reducing, which proves that the model gains more and more confidence in its predictions with the course of learning. The same thing can be said about validation accuracy, which, however, differences in natural generalization are noted and hence the result is a slightly low accuracy that is compared to the training accuracy. Initial components of the validation loss are reducing and later oscillating around average a value, which indicates the changes in the unobservable samples of data. Altogether, the trends point out that the reinforcement learning based render optimization system is stable in convergence, and the system has a high capacity of generalization. This trade-off in the learning behavior makes sure that the model is reliably able to detect rendering importance when applied to new animated scenes.

**Fig 10:**
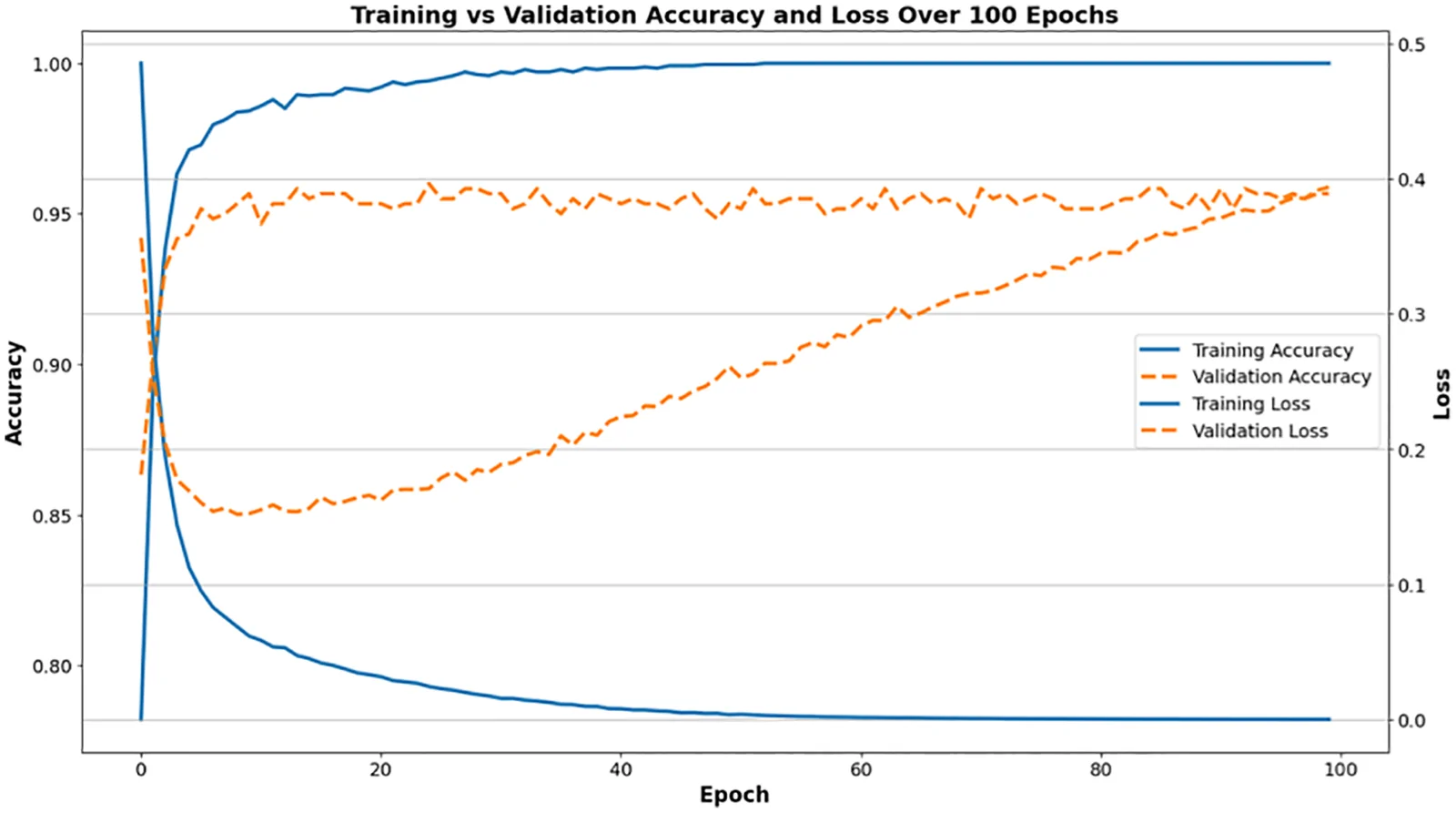
Training and Validation Performance Trends across Learning Iterations.

Fig 11 illustrates the intensity of the extracted spatial features which is used in determining the importance of rendering in animated scenes. The cells are used to show the strength of response of a given feature dimension at a given spatial index. Higher areas are more associated with high feature activations, which mean that areas are more valuable in the process of decision making of the model. The following activations depict the way the computer vision unit recognizes the structures of visual significance like object contours, motion and texture differences in the image. There are various groups of the high activation values that can be seen in other different spatial parts implying that the model does not rely on a single prominent pattern but on a number of informative features. Lower activation areas are background or less informative areas which can use less computational resources during rendering. Through these feature patterns, the reinforcement learning agent is able to rank the rendering operations of those regionally perceptually important parts. This dynamic focus system allows efficient resource allocation, which enhances the performance of the overall rendering process, as well as, the visual fidelity in systems with high complexity in the animated world.

**Fig 11:**
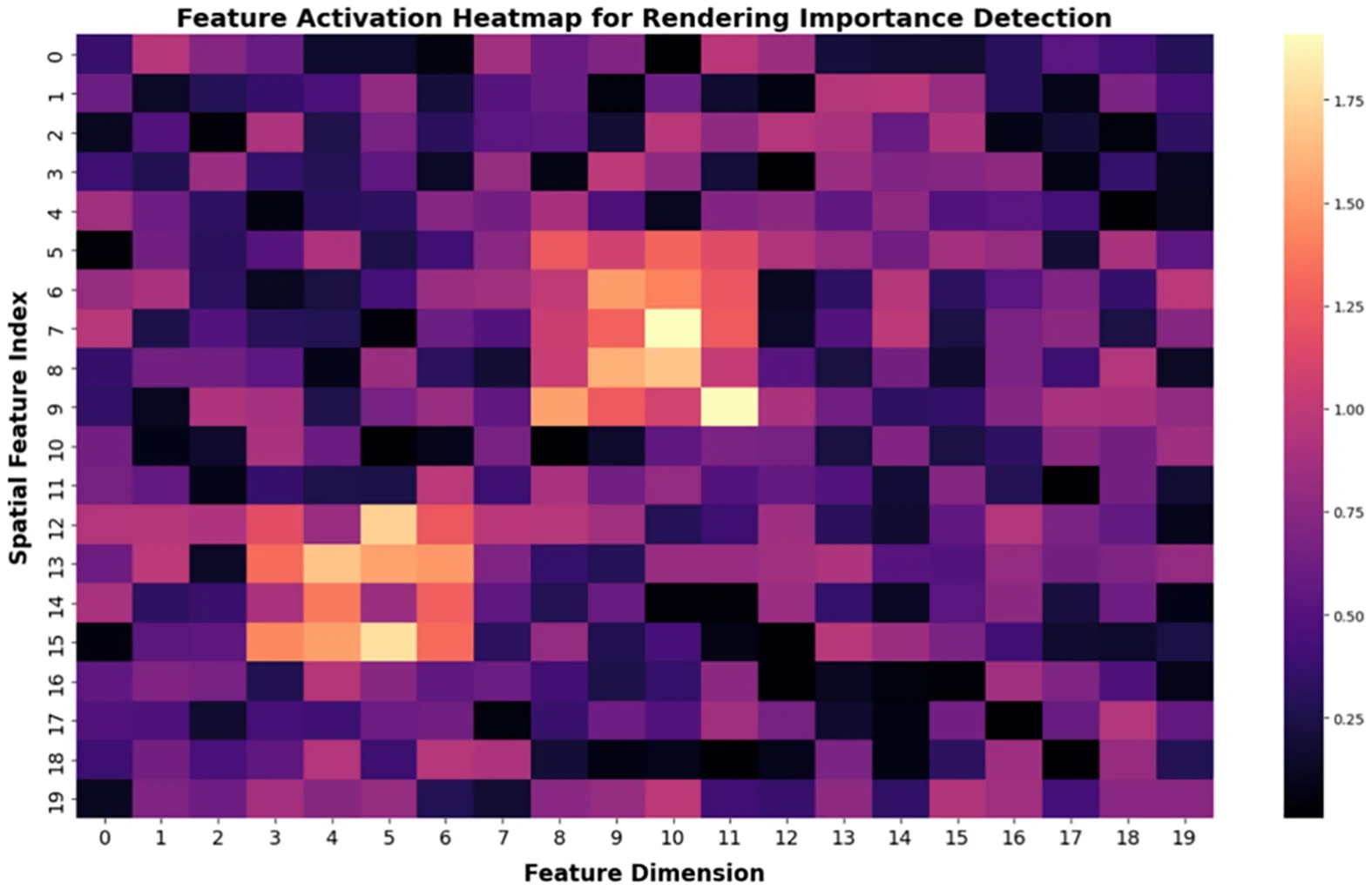
Spatial feature activation patterns for rendering importance identification.

### RL optimization for real-time animated scene rendering

Fig 12 shows the behavior of training convergence of three reinforcement learning approaches to optimization of the animated scene rendering. The offered adaptation-based variant with twin-pairs works more rapidly and attains a greater overall reward in comparison with baseline algorithms. As the agent learns better actions, the cost decreases, so the reward becomes less negative. These findings suggest that there is better policy learning, more efficient allocation of resources and greater optimization capacity in the dynamic rendering settings as training sequences get longer.

**Fig 12:**
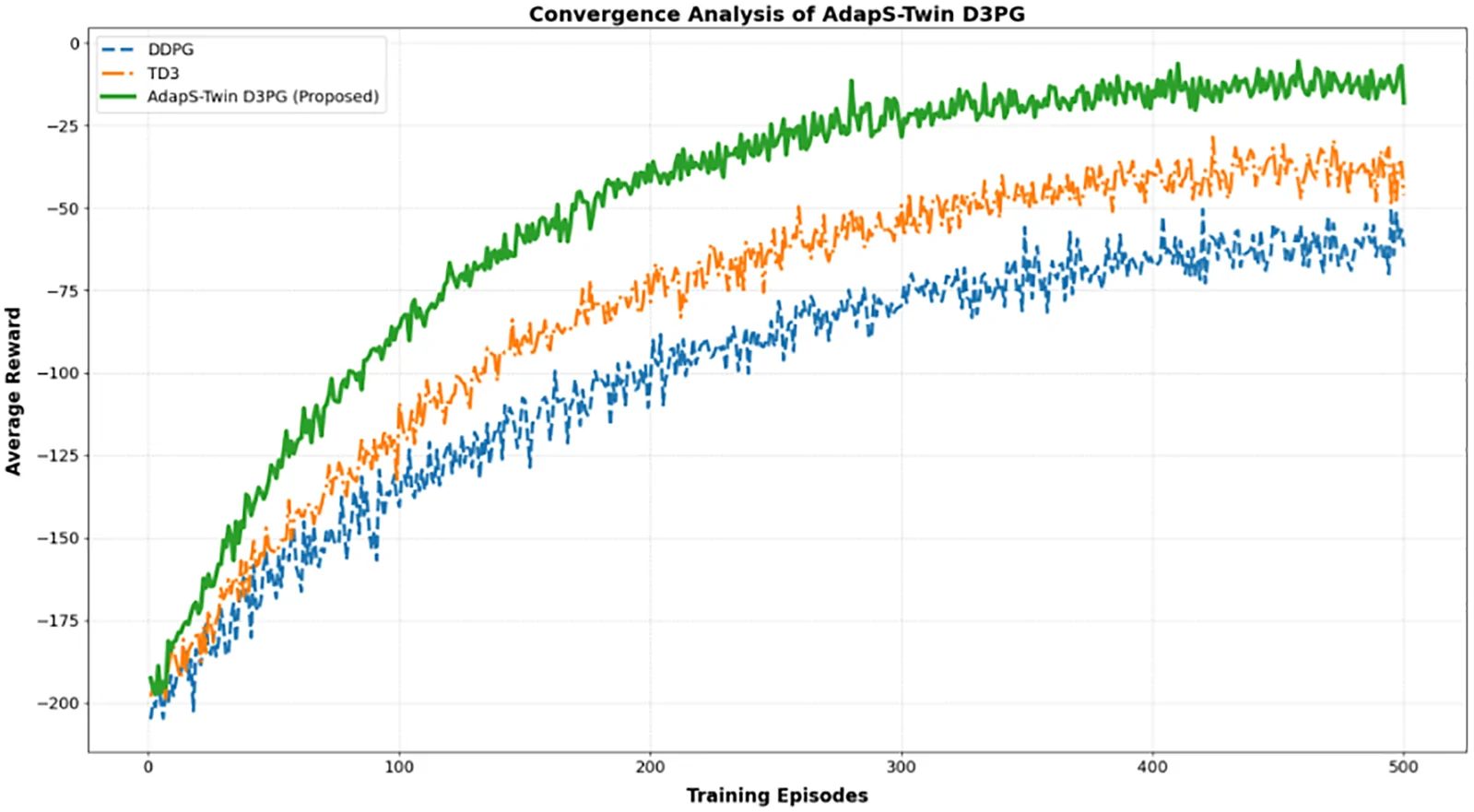
Convergence of RL Methods for Rendering Optimization Efficiency.

### RL-based rendering optimization performance comparison

The proposed AdapS-Twin D3PG clearly outperforms Twin D3PG on all metrics of RL optimization. It has more cumulative reward (276.4 vs. 185.6) and convergence faster (645 vs. 980 episodes). Variance in policy stability is diminished (0.039 vs. 0.084) which is a sign of stronger element. The exploration efficiency improves to 88.5% and the rendering accuracy to 92.7% resulting in better learning efficiency, real time rendering. Table 5 and Fig 13 show that the proposed model shows faster convergence and higher accuracy.

**Table 5:** RL optimization metrics for animated scene rendering.

RL Optimization Performance	Twin D3PG	AdapS-Twin D3PG [Proposed]
Cumulative Reward (↑)	185.6	276.4
Convergence Episodes (↓)	980	645
Policy Stability Variance (↓)	0.084	0.039
Exploration Efficiency (%) (↑)	71.3	88.5
Rendering Decision Accuracy (%) (↑)	79.8	92.7

**Fig 13:**
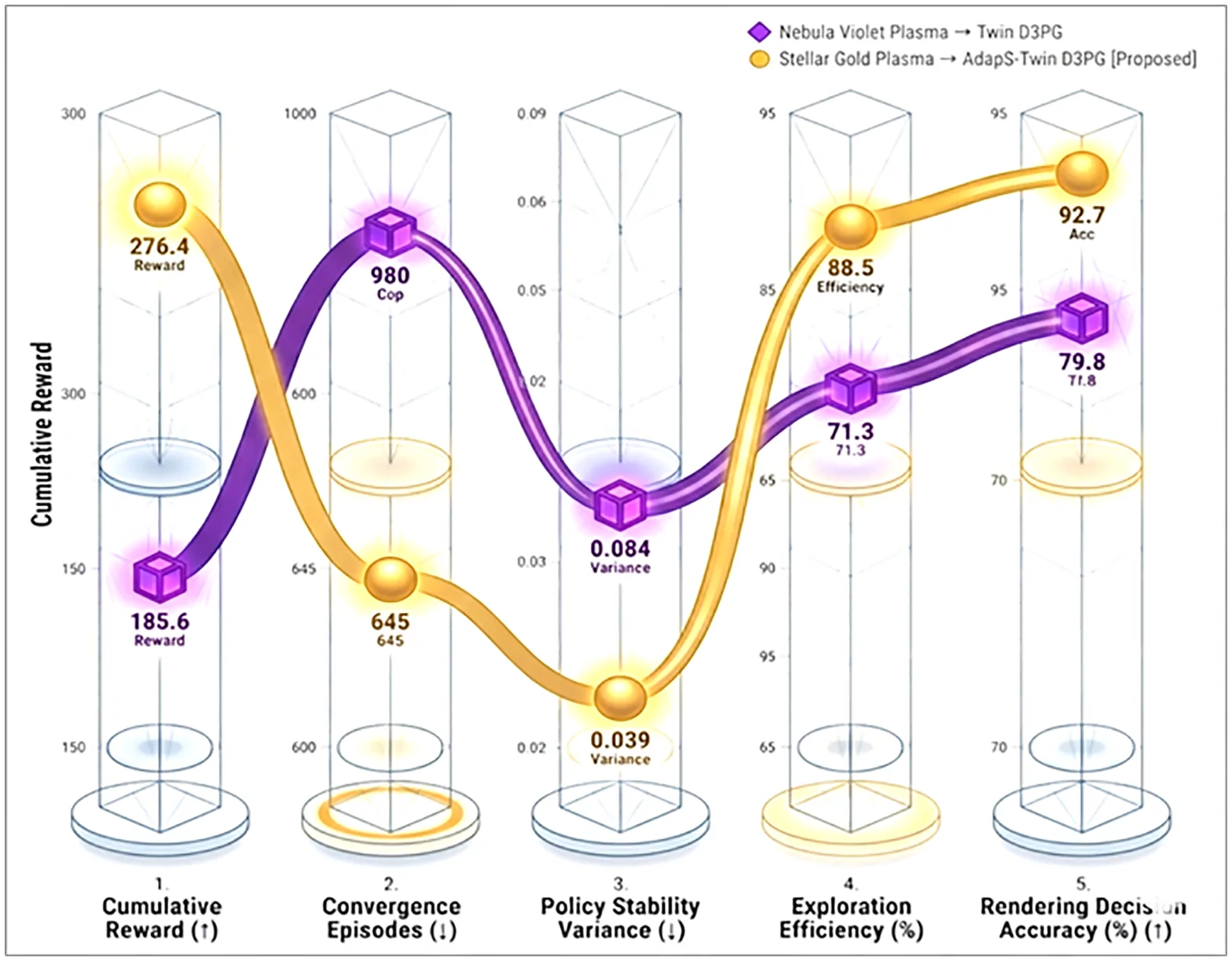
Performance comparison of twin D3PG and proposed model.

From the experimental evaluation of the proposed AdapS-Twin D3PG method, it shows the most optimal rendering for dynamic animated scenes. It outperforms the basic Twin D3PG algorithm with better results in terms of a higher cumulative reward (276.4), less time taken to converge (645 episodes), high exploration efficiency (88.5%), low variation in policy stability (0.039), and highly accurate rendering decisions (92.7%). It can be concluded from the above results that the adaptive seeker algorithm works well with Twin D3PG.

### RL-based real-time rendering optimization

The proposed AdapS-Twin D3PG works brilliantly compared to Twin D3PG in any metric. Critical Region Detection Rate of the perfectly purposive system out of 83.2% moves up to 95.1% and Dynamic Resolution Scaling Accuracy of the same activity rise out of 76.5% by 90.8%. Over-Rendering Ratio lower to 8.7% instead of 19.4% and Under-Rendering Artifact Rate lower to 6.2% instead of 15.6%. Also, the Load Balancing Efficiency increases by 74.9% to 89.6% showing a better adaptive resource distribution and more efficient real-time rendering. Table 6 and Fig 14 display a comparison of rendering accuracy and efficiency metrics.

**Table 6:** Performance comparison of adaptive rendering optimization methods.

Adaptive Resource Allocation	Twin D3PG	AdapS-Twin D3PG [Proposed]
Critical Region Detection Rate (%) (↑)	83.2	95.1
Dynamic Resolution Scaling Accuracy (%) (↑)	76.5	90.8
Over-Rendering Ratio (%) (↓)	19.4	8.7
Under-Rendering Artifact Rate (%) (↓)	15.6	6.2
Load Balancing Efficiency (%) (↑)	74.9	89.6

**Fig 14:**
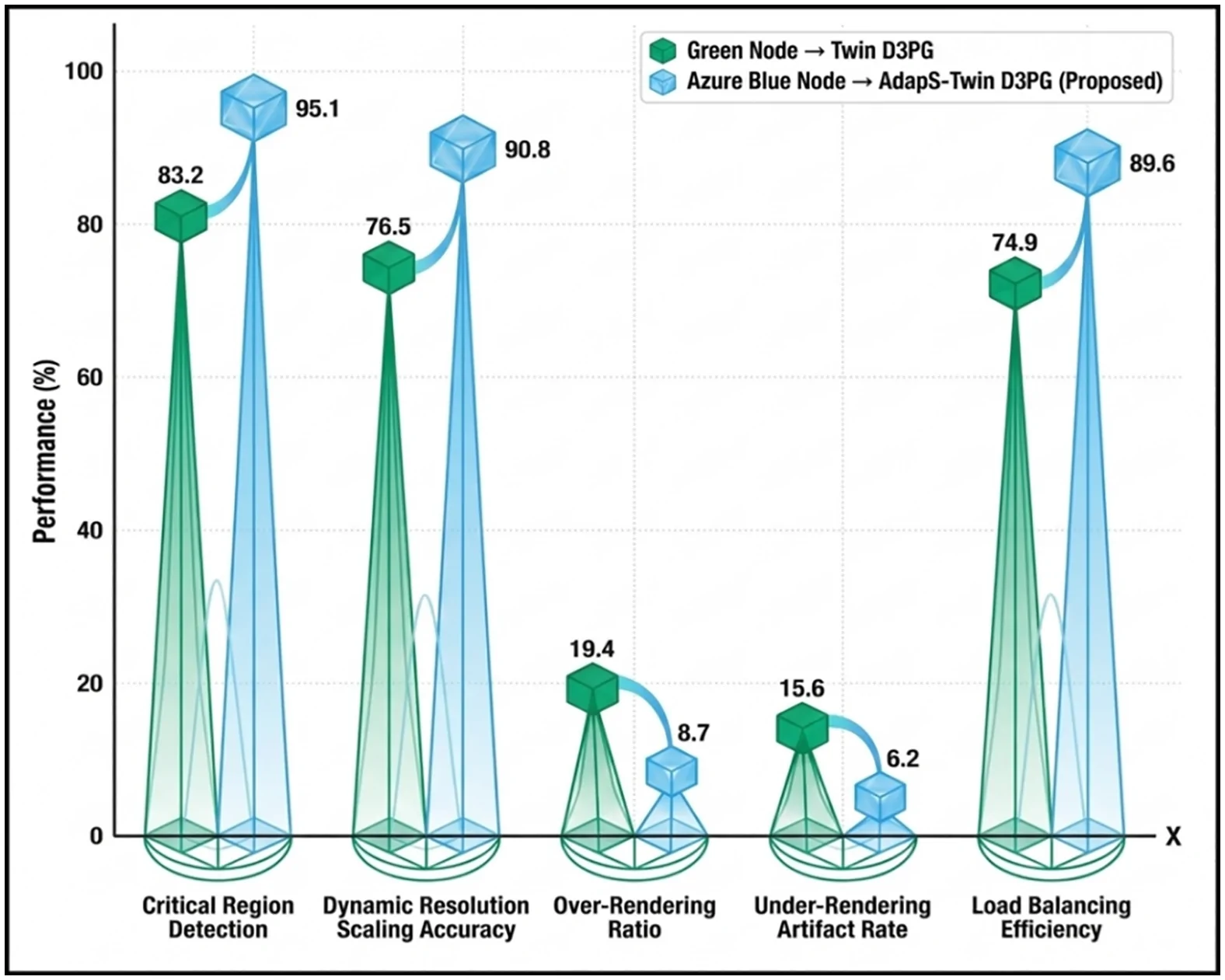
Adaptive Rendering Performance Using AdapS-Twin D3PG Model.

### Ablation study of rendering optimization framework in animated scenes

The removal of HOG led to poor discrimination of structural features, the removal of AdapS led to poor adaptive resource allocation, and the removal of Twin D3PG led to poor policy stability in the Table 7. The combination of all three components resulted in the best Accuracy, Recall, F1-Score, and AUC values.

**Table 7:** Ablation study evaluating the individual contribution of HOG, AdapS, and Twin D3PG to rendering optimization performance.

Model Configuration	Accuracy	Recall	F1 Score	AUC
Without HOG	92.84	91.23	91.47	93.56
Without AdapS	93.68	92.15	92.38	94.72
Without Twin D3PG	94.31	92.86	93.05	95.41
AdapS-Twin D3PG [Full proposed method]	95.48	93.72	93.94	96.82

### Existing real-time animation rendering and optimization methods

Existing methods in real-time rendering and optimization for animated scenes integrate simulation, deep learning, and motion transfer techniques. The Temporal-Stylistic Latent Animator (TSLA) [28] models’ temporal consistency and stylistic motion features in a latent space for controllable animation synthesis. Traditional Physical Simulation [29] ensures realism through physics-based modeling of forces, collisions, and constraints. DL (DL)-Optimized Effect Generation [29] enhances visual quality by learning rendering parameters automatically. The First Order Motion Model (FOMM) [30] enables image animation via keypoint-based motion transfer, while the Enhanced First Order Motion Model (E-FOMM) [30] improves structural consistency and identity preservation. These methods are implemented using datasets such as Human3.6M Dataset [28], Mixamo Dataset [28], Blender Dataset [29], VoxCeleb1 [30], and TaiChiHD [30] for human motion, facial animation, and realistic scene synthesis.

➢ **Accuracy** – Percentage of correctly predicted animation frames among total evaluated samples in equation (17).

$$\begin{array}{c} Accuracy=\frac{True\;Positive+True\;Negative}{True\;Positive+True\;Negative+False\;Positive+False\;Negative}\;\times \text{1}\text{0}\text{0} \end{array}$$ (17)

➢ **Recall** – Ability to correctly detect relevant optimized rendering outputs from the total actual positives in equation (18).

$$\begin{array}{c} Recall=\frac{True\;Positive}{True\;Positive+False\;Negative}\times \text{1}\text{0}\text{0} \end{array}$$ (18)

➢ **F1 Score** – Harmonic mean of Precision and Recall, indicating balanced performance evaluation in equation (19).

$$\begin{array}{c} F\text{1}=\frac{\text{2}\times Precision\times Recall}{Precision+Recall}\times \text{1}\text{0}\text{0} \end{array}$$ (19)

➢ **AUC (Area under the Curve)** – Measures model discrimination ability across classification thresholds in equation (20).

$$\begin{array}{c} AUC=\int_{\text{0}}^{\text{1}}True\;Position\;Rate(False\;Position\;Rate)d(False\;Position\;Rate) \end{array}$$ (20)

➢ **PSNR** – Quantifies reconstructed frame quality compared to ground truth reference images in equation (21). Where ${\text{MAX}}^{\text{2}}$: Maximum pixel value and MSE: Mean Squared Error.

$$\begin{array}{c} PSNR=\text{1}\text{0}{log}_{\text{1}\text{0}}(\frac{{MAX}^{\text{2}}}{MSE}) \end{array}$$ (21)

➢ **FID (Fréchet Inception Distance)** – Measures distribution similarity between generated frames and real images for realism assessment in equation (22).$\mu_{\text{r}},\mu_{\text{g}}$: Mean feature vectors, $\Sigma_{\text{r}}\Sigma_{\text{g}}$: Covariance matrices, and Tr: Matrix trace operator.

$$\begin{array}{c} FID={\left|\left|\mu_{r}-\mu_{g}\right|\right|}^{\text{2}}+Tr(\Sigma_{r}+\Sigma_{g}-\text{2}{\left(\Sigma_{r}\Sigma_{g}\right)}^{\frac{\text{1}}{\text{2}}}) \end{array}$$ (22)

➢ **Rendering Time (Seconds)** – Total computational time required to produce optimized animated frames. Equation (23)${\text{ t}}_{\text{i}}$: Time to render frame i, $T_{render}$ is the total time required for scene rendering.and N: Total number of frames.

$$\begin{array}{c} T_{render}=\sum_{i=\text{1}}^{N}t_{i} \end{array}$$ (23)

➢ **LPIPS (Learned Perceptual Image Patch Similarity)** – Perceptual similarity metric; lower values indicate higher visual similarity in equation (24). Where x,y: Compared images,LPIPS(x,y): Measures perceptual similarity between images, $\Phi_{\text{l}}$: Deep network features at layer l, and $\omega_{\text{l}}$: Layer weight.

$$\begin{array}{c} LPIPS(x,y)=\sum_{l}\omega_{l}.{\left|\left|\Phi_{l}(x)-\Phi_{l}(y)\right|\right|}_{\text{2}}^{\text{2}} \end{array}$$ (24)

➢ **NeRF-NQA (Neural Radiance Field No-Reference Quality Assessment)** – No-reference metric; higher values indicate better rendering quality. In equation (25), ${\text{F}}_{\text{NeRF}}$: Extracted NeRF-based rendering features, f(.): Learned quality prediction function, and Q: Predicted quality score.

$$\begin{array}{c} Q=f(F_{NeRF)} \end{array}$$ (25)

### RL-based real-time animated scene optimization

The table compares TSLA [28] and the proposed AdapS-Twin D3PG across Human3.6M, Mixamo, and ATD_12 datasets. On Human3.6M, TSLA achieves 90.84 accuracy, 88.52 recall, 87.91F1, and 91.27AUC, while AdapS-Twin D3PG improves to 93.12 accuracy, 91.45 recall, 90.88 F1, and 94.36 AUC. On Mixamo, TSLA records 89.73 accuracy, 86.39 recalls, 86.54 F1, and 90.88 AUC, whereas the proposed method achieves 92.04 accuracy, 89.27 recall, 89.61 F1, and 93.18 AUC. On ATD_12, TSLA scores 88.64 accuracy, 85.71 recall, 85.93 F1, and 89.76 AUC, while AdapS-Twin D3PG reaches 95.48 accuracy, 93.72 recall, 93.94 F1, and 96.82 AUC. Table 8 and Fig 15 show that the proposed model consistently outperforms TSLA across all datasets.

**Table 8:** Performance comparison across Human3.6M, Mixamo, and ATD_12 datasets.

Method	Human3.6M Dataset				Mixamo Dataset				ATD_12 Dataset			
	Accuracy	Recall	F1 Score	AUC	Accuracy	Recall	F1 Score	AUC	Accuracy	Recall	F1 Score	AUC
TSLA [28]	90.84	88.52	87.91	91.27	89.73	86.39	86.54	90.88	88.64	85.71	85.93	89.76
AdapS-Twin D3PG [Proposed]	93.12	91.45	90.88	94.36	92.04	89.27	89.61	93.18	95.48	93.72	93.94	96.82

**Fig 15:**
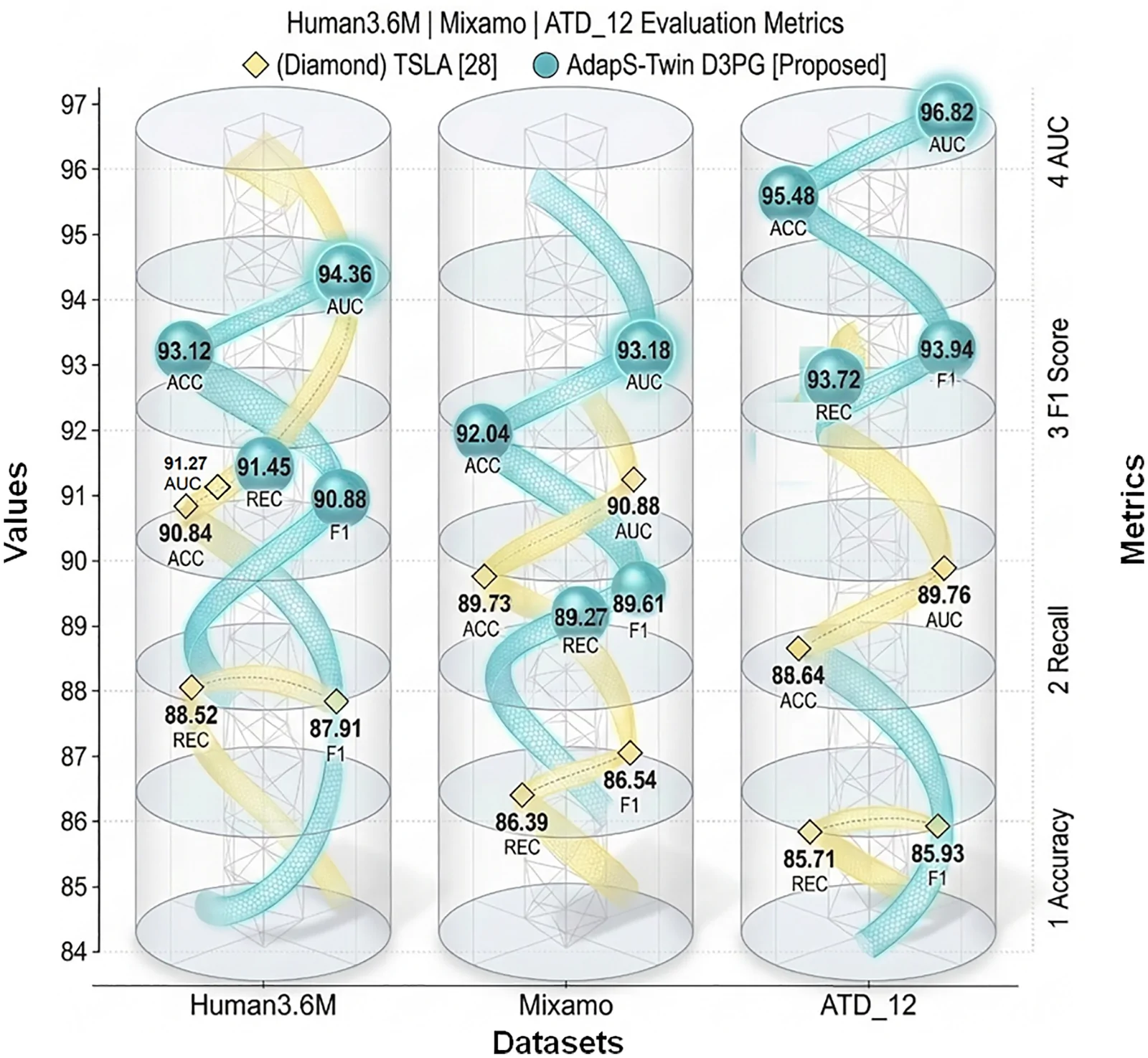
AdapS-Twin D3PG Performance across Three Benchmark Datasets.

### Performance comparison of animated scene rendering techniques

The table compares rendering quality and efficiency across two datasets. On the Blender Dataset, Traditional Physical Simulation achieves 30.2 dB PSNR, 23.4 FID, and 20.8 s rendering time, while DL-Optimized Effect Generation improves to 34.5 dB PSNR, 18.7 FID, and 6.7 s. The proposed AdapS-Twin D3PG further enhances performance with 36.8 dB PSNR, 16.2 FID, and 5.4 s. On the ATD_12 Dataset, Traditional Simulation records 31.6 dB PSNR, 22.1 FID, and 18.9 s; DL-Optimized reaches 35.9 dB, 17.3 FID, and 6.1 s; AdapS-Twin D3PG achieves 39.4 dB, 13.8 FID, and 4.2 s. Table 9 and Fig 16 display PSNR, FID, and time comparison for animated scene methods.

**Table 9:** Quantitative evaluation of animated scene rendering methods on datasets.

Method	Dataset	PSNR (dB)	FID	Rendering Time (seconds)
Traditional Physical Simulation [29]	Blender Dataset	30.2	23.4	20.8
DL-Optimized Effect Generation [29]		34.5	18.7	6.7
AdapS-Twin D3PG [Proposed]		36.8	16.2	5.4
Traditional Physical Simulation [29]	ATD_12 Dataset	31.6	22.1	18.9
DL-Optimized Effect Generation [29]		35.9	17.3	6.1
AdapS-Twin D3PG [Proposed]		39.4	13.8	4.2

**Fig 16:**
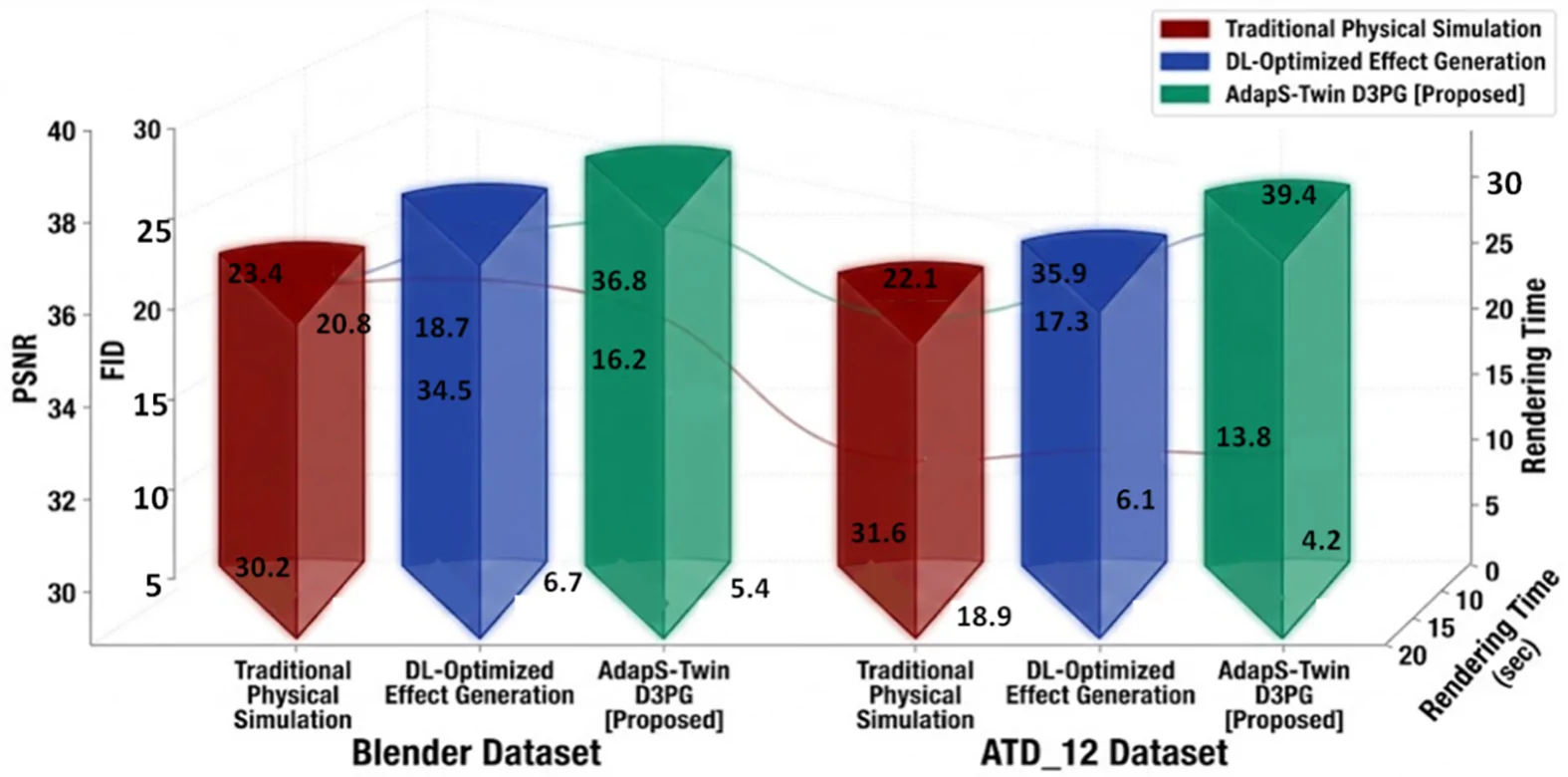
Animated Scene Rendering Performance across Blender and ATD_12 Datasets.

### Reinforcement learning–based real-time animated scene rendering evaluation

The table compares E-FOMM, FOMM, and the proposed AdapS-Twin D3PG across VoxCeleb1, TaiChiHD, and ATD_12 datasets using LPIPS (↓) and NeRF-NQA (↑). For VoxCeleb1, E-FOMM records 0.045 and 0.92, FOMM 0.061 and 0.88, while AdapS-Twin D3PG achieves 0.032 and 0.96. On TaiChiHD, values are 0.038/0.95 (E-FOMM), 0.055/0.89 (FOMM), and 0.028/0.97 (Proposed). For ATD_12, results are 0.034/0.96, 0.049/0.91, and 0.021/0.985, respectively. Overall, the proposed model consistently delivers the lowest LPIPS and highest NeRF-NQA, demonstrating superior rendering fidelity and perceptual quality across all datasets. Table 10 and Fig 17 illustrate that the proposed method achieves the lowest distortion and highest quality.

**Table 10:** Quantitative comparison of rendering performance across benchmark datasets.

Method	Dataset	LPIPS ↓	NeRF-NQA ↑
E-FOMM [30]	VoxCeleb1	0.045	0.92
	TaiChiHD	0.038	0.95
	ATD_12 Dataset	0.034	0.96
FOMM [30]	VoxCeleb1	0.061	0.88
	TaiChiHD	0.055	0.89
	ATD_12 Dataset	0.049	0.91
AdapS-Twin D3PG [Proposed]	VoxCeleb1	0.032	0.96
	TaiChiHD	0.028	0.97
	ATD_12 Dataset	0.021	0.985

**Fig 17:**
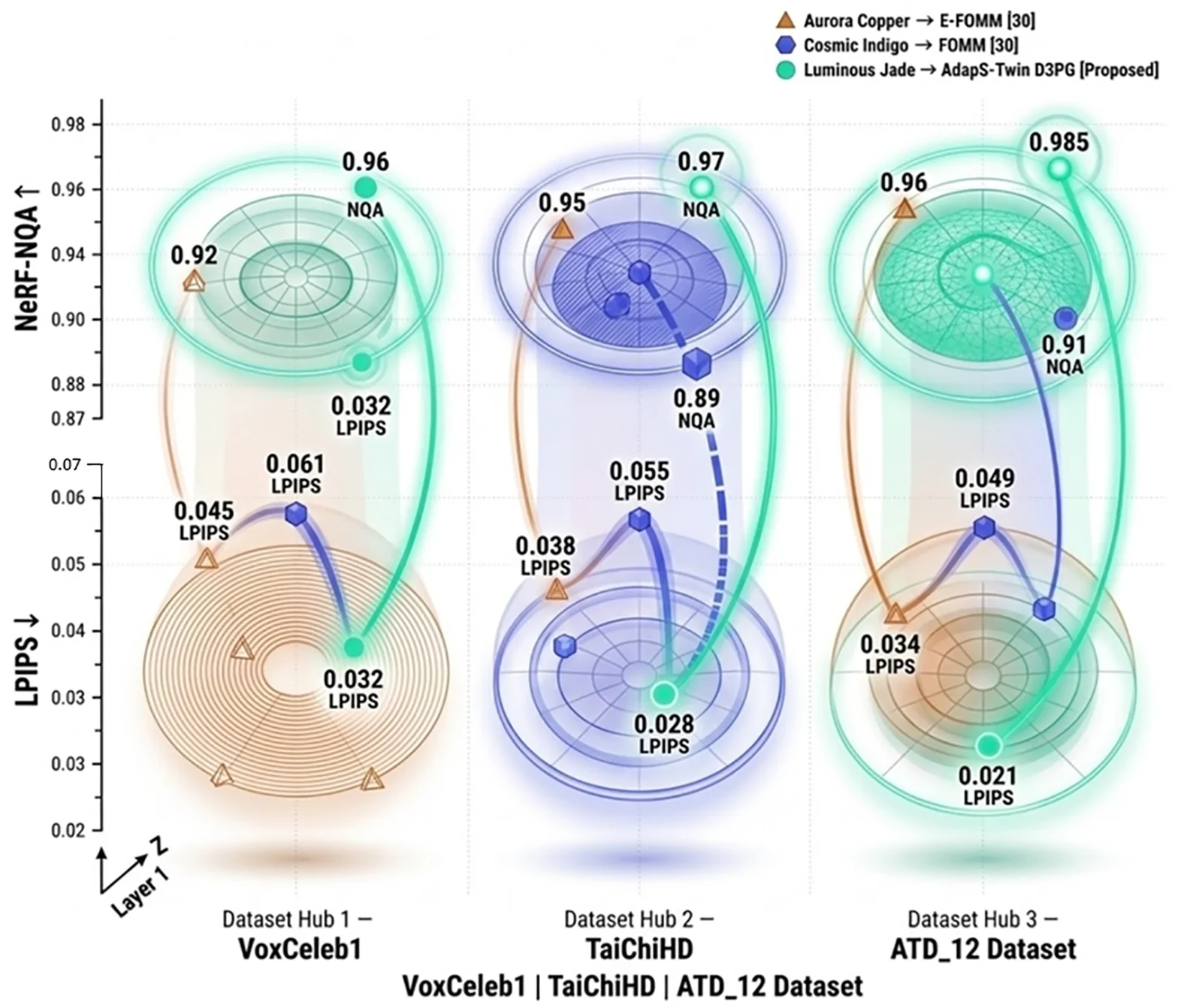
Performance Comparison of Rendering Methods Using LPIPS and NeRF-NQA.

The comparison between AdapS-Twin D3PG and Traditional Physical Simulation, DL-Optimized Effect Generation, E-FOMM, and FOMM is shown in Table 11, using the ATD_12 data set. The proposed algorithm performed the best among all methods 60.8 FPS, 39.4 dB visual quality, 0.978 SSIM value, 95.1% critical region detection rate, 90.8% accurate dynamic resolution scaling, 276.4 cumulative reward, and converged at 645 episodes.

**Table 11:** Existing methods and proposed method were retrained on the ATD_12 Dataset.

Methods	Frame rate	Visual quality	SSIM	Critical Region Detection Rate	Dynamic Resolution Scaling Accuracy	Cumulative Reward	Convergence Episodes
TSLA	42.8	34.8	0.926	81.7	79.1	165.3	1088
Traditional Physical Simulation	28.4	31.6	0.892	72.8	68.9	102.6	1420
DL-Optimized Effect Generation	48.7	35.9	0.931	84.3	81.2	176.8	1015
E-FOMM	52.9	37.6	0.952	89.4	86.7	224.9	812
FOMM	44.6	36.2	0.941	86.8	83.5	201.7	928
AdapS-Twin D3PG [Proposed]	60.8	39.4	0.978	95.1	90.8	276.4	645

Comparison of AdapS and AdapS-Twin D3PG using the paired t-test is shown in Table 12. There were statistically significant improvements in Accuracy, Recall, F1 Score, and AUC for the proposed model, with the presence of positive mean difference, high t-value, low p-value, and narrow 95% confidence interval.

**Table 12:** Paired t-test statistical comparison between AdapS and AdapS-Twin D3PG performance metrics.

Metrics	AdapS (Mean)	AdapS-Twin D3PG (Mean)	Mean Difference	t-value	p-value	95% CI
Accuracy	94.18	95.48	1.30	6.84	0.0019	0.78–1.82
Recall	92.34	93.72	1.38	6.57	0.0023	0.83–1.93
F1 Score	92.61	93.94	1.33	6.71	0.0021	0.79–1.87
AUC	95.13	96.82	1.69	7.26	0.0013	1.08–2.30

## Discussion

The Real-Time Rendering and Optimization System of Animated Scenes is a system that unites RL with CV, being able to dynamically modify rendering parameters based on the complexity of the scenes. The system sacrifices the computational overhead by determining the visually important areas and assigning the computer resources dynamically, which enhances visual fidelity. This clever prioritization allows its use to generate frames efficiently and is stable even when in a very dynamic animation environment. Conventional Physical Simulation techniques involve the use of dense physics-based calculations that add time to processing and restrict real-time scalability in complicated animation settings [29]. Deep learning (DL)-based effect generation methods are more realistic and require large training datasets and lots of GPU power, which makes them hard to use in real-time production systems [29]. Temporal Sequence Learning Approaches (TSLA) ensure maintenance of temporal continuity, but they tend to be inefficient at allocating rendering resources in the busy or difficult scenes [28]. First Order Motion Models (FOMM) permit motion transfer of images but are vulnerable to visual artifacts with large pose changes and complicated backgrounds [30]. Even though Enhanced-FOMM (E-FOMM) can enhance motion estimation accuracy, it does not offer adaptive rendering control and efficient management of the use of computational resources to optimize scenes in real time [30]. The proposed AdapS-Twin D3PG technique deals with these issues by training adaptive rendering policies, which rank significant visual regions. This has found extensive usefulness in real-world applications like real-time video game graphics, virtual reality simulations, animated film production and interactive training systems where quality visual performance with limited computation resources is needed.

The performance of the intelligent rendering framework in question indicates its great flexibility within various animated scenes and benchmark rendering datasets, due to the ability to provide good performance both in terms of rendering quality and speed. The evaluation of the model’s performance using such datasets as Human3.6M, Mixamo, ATD-12, Blender, VoxCeleb1, and TaiChiHD datasets proves its greater Accuracy, Recall, F1-score, AUC, PSNR, LPIPS, NeRF-NQA, FID, and rendering time compared to other rendering methods.

## Conclusion

RL and CV are combined to create a real-time rendered, optimized procedure of lighting, textures, and geometry in an animated rendering. The system improves the quality of rendering, lowers the cost, and offers effective and high-quality visual execution through learning of scene dynamics and visual significance. A real-time adaptive rendering system, which combines CV and RL to generate the animated scenes in the most optimized way. The ATD-12 dataset which contained 10,000 animation image triplets used in motion analysis tasks was collected. SavitzkyGolay preprocessing and Min Max normalization were used to improve consistency of data and minimize noise. With HOG used in the structural scene extraction, the proposed AdapS-Twin D3PG model was optimized to intelligently sharpen critical areas, thereby producing a better visual image and lowering the computational cost of image production. The suggested AdapS-Twin D3PG has 95.48 accuracy, 93.72 recall, 93.94 F1-score, and 96.82 AUC. It achieves 39.4 dB PSNR, 13.8 FID, and 4.2 seconds rendering time, and provides high fidelity with 0.021 LPIPS and 0.985 NeRF-NQA, which is highly efficient. The system is prone to huge computational loads, reward complexity, training instability and reliance on large annotated datasets. On low-end hardware, latency on real-time deployment might be problematic and generalization to different animated scenes is limited. By training lightweight models, adaptive reward Shaping, transfer-based learning, and edge-based optimization, the model may be scaled more, be more robust, cross-scene flexible, and more performance efficient. The proposed method has not been validated on real-time rendering engines or practical rendering pipelines, limiting its practical applicability and deployment feasibility in real-world animation rendering environments.
